# Development of isothermal nucleic acid amplification technologies for rapid detection of Porcine Enterovirus-G

**DOI:** 10.1371/journal.pone.0326700

**Published:** 2025-07-02

**Authors:** Sarshti Kaushik, Sushila Maan, Kanisht Batra, Swati Sindhu, Vijay Kadian, Aman Kumar

**Affiliations:** 1 Lala Lajpat Rai University of Veterinary and Animal Sciences (LUVAS), Hisar, Haryana, India; University of Florida Tropical Research and Education Center, UNITED STATES OF AMERICA

## Abstract

Porcine enterovirus G (PEV-G) presents a considerable threat to the swine industry, causing a range of diseases that include diarrhea, encephalomyelitis, reproductive disorders, and respiratory infections. Conventional diagnostic approaches, such as virus isolation and RT-PCR, are frequently labor-intensive and reliant on specialized equipments. Therefore, there is an immediate need for isothermal nucleic acid amplification techniques-specifically, Recombinase Polymerase Amplification (RPA) and Polymerase Spiral Reaction (PSR) that offer rapid, sensitive, and field-deployable detection of PEV-G. In this study, we successfully developed and optimized two isothermal nucleic acid amplification assays namely RPA/RT-RPA and PSR/RT-PSR to detect PEV-G in swine populations in Haryana. Primers were specifically designed to target the polyprotein region of PEV-G for both assays. Optimal conditions regarding temperature, incubation time, primer concentration, and magnesium ion concentration were established. The RPA assay demonstrated a sensitivity of 1.417 × 10⁴ copies with a detection time of just 20 minutes. The PSR assay exhibited a lower sensitivity of 2.3 x 10^5^ copies in comparison to RPA assay in gel based detection system and required 2.5 hours for detection. Both assays showed exceptional specificity for PEV-G, with no observable cross-reactivity with other related porcine viruses. Additionally, visual detection using Picogreen dye provided a practical solution for field use, with limits of detection of 14 copies for RPA and 2.3 copies for PSR. Validation on 100 archived field samples showed that isothermal assays have comparable sensitivity to conventional PCR. This study underscores the potential of RPA and PSR as effective and cost-efficient diagnostic tools, enabling timely and precise detection of PEV-G in both laboratory and field contexts. Such advancements are vital for improving disease management strategies and reducing economic losses within the swine industry.

## Introduction

Pig farming represents a vital avenue for meat production in developing countries like India, where pigs are a prolific source of protein, significantly benefiting the livestock sector [1]. However, the industry frequently faces viral disease outbreaks that result in substantial economic losses. A range of diseases linked to subclinical infections in swine herds is caused by various pathogens, including Porcine enteroviruses (PEV), Porcine Toroviruses (PToV), Porcine Sapelovirus (PSV), Porcine Bocavirus (PBoV), and Porcine Kobuvirus (PKBV) [2]. These viral infections can lead to serious health issues in pigs, manifesting as diarrhea, respiratory problems, polioencephalomyelitis, skin lesions, and reproductive disorders, with outcomes influenced by the viral strain, the animal’s immune status, and prevailing environmental conditions.

The *Picornavirales* order encompasses a diverse group of RNA viruses that infect various hosts. According to the International Committee on Taxonomy of Viruses (ICTV) [3], this order consists of five families: *Dicistroviridae, Iflaviridae, Secoviridae, Picornaviridae*, and *Marnaviridae*. Among these, Porcine Enterovirus-G (PEV-G), belonging to the *Picornaviridae* family, is classified into three genetic groups: PEV types 1–7 and 11–13 (*Teschovirus*), PEV-8 (*Sapelovirus*), and PEV-9 and 10 (*Porcine Enterovirus* G). Genetic studies have identified at least 20 distinct PEV-G types (EV-G 1–20) [4,5]. These viruses infect a multitude of hosts, not only including pigs (species G) but also humans (species A–D), cattle (species E and F), camels (species I), rodents (species K), and non-human primates (species A, B, D, H, J, and L) [6–8]. PEV-G is characterized as a small, non-enveloped virus with a spherical capsid approximately 30 nm in diameter, featuring a single-stranded positive-sense RNA genome ranging from 7 to 8.8 kb. Its genome is organized into key regions: a 5’ untranslated region (UTR), P1, P2, P3, and a 3’ UTR, where the P1 region encodes four structural proteins (VP1-VP4), and the P2 and P3 regions produce various precursor proteins that are cleaved into seven non-structural proteins via proteases [9,10].

Conventional diagnostic methods, such as virus isolation and serological techniques, present several limitations, including time consumption, labor intensity, and sensitivity levels ranging from 62.6% to 75% [11,12]. Serological tests like ELISA and neutralization assays also face issues such as cross-reactivity and the need for serotype-specific requirements [13], which render them less suitable for rapid or large-scale testing. In contrast, molecular diagnostic tools such as RT-PCR and qPCR offer more rapid and precise identification of the virus, generally delivering results within three hours [14,15]. However, their reliance on advanced equipment and skilled personnel can limit their use in primary care or resource-limited environments.

To address these challenges, isothermal nucleic acid amplification technologies have been introduced for point-of-care testing (POCT). Recombinase Polymerase Amplification (RPA) is a user-friendly and rapid method that operates effectively at temperatures of 37–42°C, making it suitable for field applications [16]. Similarly, Polymerase Spiral Reaction (PSR) provides quick and highly specific results at a constant temperature of 60–67°C [17]. These innovative techniques eliminate the need for costly thermocyclers, thus enhancing accessibility in resource-constrained settings while enabling efficient disease detection and management in pig farming. Implementing these advanced diagnostic tools will not only help mitigate the impact of viral diseases on the swine industry but also contribute to improving food security and economic stability in developing regions.

## Materials and methods

### Ethics statement

The samples used in this study were obtained from a naturally infected animal in the field, by qualified veterinarians, as a part of normal veterinary care and diagnostic testing procedures. The ethical approval was taken for this study from the Institutional Animal Ethics Committee (IAEC), registered as 1669/GO/ReBiBt/S/12/CPCSEA dated 6.12.2012 in 25^th^ meeting held on 28^th^ October, 2022.

### Sample details and viral RNA extraction

A total of 100 archived samples collected from different organized and unorganized piggery of Haryana, India (29.0588° N, 76.0856° E), were used in this study. Faecal and nasal swabs were collected from all age group of animals. Faecal samples were collected in stool collection vial while nasal samples were collected with the help of sterile swabs and stored at −20ºC till further processing. Faecal and nasal samples were dissolved in PBS (10% w/v) and debris were separated by centrifugation at 10,000xg for 10 minutes. Supernatant was collected in separate microcentrifuge tube and stored at −20ºC for further processing. The viral RNA was extracted by Trizol method in combination with QIAmp viral RNA mini kit (Qiagen). The extracted viral RNA was quantified and purity was tested with Nanodrop 2000 (Thermo Fisher Scientific Inc.).

### Primer design

The primers for PEV-G were designed internally, focusing on the polyprotein region of the viral genome. For the RPA assay, primers were synthesized to target the same polyprotein region, which translates into non-structural proteins 2A and 2B [18]. The forward primer (PEV-G/3424-3443F) sequence is 5’-TACAACAGAGATCTTCTTGT-3’, while the reverse primer (PEV-G/3602-3581R) sequence is 5’-TGGTAYCTAGCYGGGTAGTATT-3’. These primers were designed to produce a 179 bp amplicon in the RPA reactions. Both primers were synthesized by Sigma-Aldrich Chemical Pvt. Ltd. Bangalore, India and underwent HPLC purification.

PSR primers were synthesized to target the polyprotein region of the genome, which later translates into non-structural protein 2C. The forward primer (PEV-G/4255-4276F) sequence is 5’-ttgaaattgaaaccattGARCATTCCTGYCCAACWACAG-3’, while the reverse primer (PEV-G/4415-4395R) sequence is 5’-ttaccaaagttaaagttTTGCTCTTGAACTGTATGTAT-3’, designed to produce a 195 bp amplicon. An adapter oligonucleotide sequence of exogenous origin was added to the 5’ end of the primers (indicated by lower font letters), ensuring that its melting temperature (Tm) was 5°C lower than that of the primer sequences. The primers were synthesized by Sigma Aldrich Chemical Pvt. Ltd. Bangalore, India and were desalted.

### Preparation of cDNA

The archived PEV-G positive sample (IND2021/ABT278) was used for cloning and transformation experiments. The recombinant plasmids obtained from the PCR products cloning served as positive controls for each assay development.

#### Using GoScript™ cDNA kit (Promega).

The cDNA was synthesized using GoScript™ cDNA kit (Promega, A5003). As per the manufacture’s protocol, 20 μl of reaction mixture was prepared by adding 1 μl of random primer, 5 μl RNA (150 ng/μl) and 2 μl nuclease free water (NFW). The mixture was heated at 70ºC for 5 min and snap chilled on ice for 2 min. Buffer mixture consisting of 4 μl of 5x reaction buffer, 1.2 μl MgCl_2_ (25mM), 1 μl PCR nucleotide mix, 0.5 μl Ribonuclease inhibitor, 1 μl Reverse Transcriptase and 4.3 μl NFW was added to 8 μl of snap chilled mixture. The primer mixture and buffer mixture were mixed properly by vortexing the tube. cDNA was prepared by incubating the RT and primer mix at 25ºC for 5 min, followed by incubation at 42°C for 60 min and final incubation at 72°C for 15 min. The cDNA was stored in −20° for further use.

#### Using super script TMIV reverse transcriptase.

PEV-G RNA was subjected to cDNA synthesis using SuperScript^TM^IV Reverse Transcriptase (Invitrogen by Thermo Fischer Scientific, cat no. 01288215) according to manufacturer’s instructions. As per the manufacture’s protocol, 20 μl of reaction mixture was prepared by adding 1 μl of both specific primers, 1 μl RNA (10ng/μl), 1 μl of dNTP (10mM) and 9 μl NFW. The mixture was heated at 65ºC for 5 min and snap chilled on ice for 1 min. Buffer mixture consisting of 4 μl of 5x SSIV buffer, 1 μl Dithiothreitol (DTT) (10mM) (Invitrogen), 1 μl Ribonuclease inhibitor (Promega), 1 μl Superscript reverse transcriptase was added to 13 μl of snap chilled mixture. The primer mixture and buffer mixture were mixed properly by vortexing the tube followed by pulse centrifugation to bring the reagents down in the tube. Then, the combined mixture was then incubated at 50ºC for 10 min followed by inactivation of reaction at 80ºC for 10 min. The cDNA was stored in −20°C for further use.

### PCR amplification and cloning

The PCR-amplified products obtained from the IND2021/ABT278 sample, using primers for RPA (179 bp) and PSR (195 bp), were separated using agarose gel electrophoresis (AGE). The gel-purified products, processed with the QIAquick Qiagen kit, were subsequently cloned using the CloneJET PCR cloning kit from Thermo Scientific and transformed into the *E. coli* DH5-alpha strain. Recombinant clones were isolated using the Zymopure miniprep kit, and positive clones were assessed by AGE and further verified by PCR with specific primers for both assays. These clones were utilized as positive controls for optimizing the RPA and PSR assays.

### RPA assay optimization for PEV-G

RPA reactions were conducted following the manufacturer’s instructions the TwistAmp^TM^ Basic Kit (TwistDx, Inc., Maidenhead, United Kingdom; catalog number TABAS03KIT). The kit components include a rehydration buffer, a pellet containing enzymes, and magnesium acetate. As per the manufacturer’s guidelines, the enzyme pellet was dissolved in the rehydration buffer. The recombinant plasmid served as a positive control with gene-specific primers. The reaction mixture, as specified in the kit, consisted of the following components: 2.4 µl of each primer (10 µM), 29.5 µl of rehydration buffer, 1.32 µl of nuclease-free water (NFW), 12 µl of plasmid DNA template/cDNA, and 2.48 µl of magnesium acetate added last to initiate the RPA reaction.

For the optimization of the RPA, various parameters were evaluated, including reaction temperature, duration, forward primer concentration, reverse primer concentration, and magnesium acetate concentration. The optimal reaction temperature was determined by testing conditions at 35°C, 37°C, 39°C, 41°C, 43°C, and 45°C for 20 minutes, using a known concentration of plasmid DNA as the template. The optimal reaction time was assessed by varying the reaction duration to 5, 10, 15, 20, 25, and 30 minutes. The optimal concentration ranges for both forward (0.24–0.96 µM) and reverse primers (0.24–0.96 µM) were established through checkerboard titration, testing a total of 16 combinations. To maximize the efficiency of the developed assay, various magnesium acetate concentrations (10 mM, 12 mM, 14 mM, 16 mM, 18 mM, and 20 mM) were also evaluated.

### Development of one step RT-RPA

One step RT-RPA assay was developed for PEV-G. Two different reverse transcriptase enzymes Super Script^TM^IV Reverse Transcriptase (Invitrogen), and MMLV reverse transcriptase (Promega) were tested for development of this assay. The reaction mixture contains 2.4 μl each of forward primer (0.96 µM), 2.4 μl each of reverse primer (0.72 µM), 29.5 µl of rehydration buffer, 4 μl of DTT (10 mM), 2 μl of Superscript IV RT/MMLV RT (200 U/µL), RNA template 7.22 µl and to initiate the RPA reaction 2.48 µl of magnesium acetate was added in the end.The reaction mixture was subject to incubation at 41ºC for 20 minutes. The results were visualised using 2.5% agarose gel electrophoresis as well as visual detection using Picogreen dye.

### PSR assay optimization for PEV-G

The PSR of the PEV-G genome was conducted using isothermal amplification buffer (New England Biolabs, NEB), Betaine (Sigma-Aldrich Chemical Pvt. Ltd. Bangalore, India), and *Bst* polymerase enzyme (New England Biolabs). An isolated plasmid was utilized as a positive control, and the PSR for PEV-G was optimized under various conditions using in-house designed primers. The PSR reaction mixture was prepared at a volume of 25 µl, comprising the following components: 2.5 µl of Isothermal Buffer (10X), 2 µl of MgSO₄ (50 mM), 5 µl of Betaine (5 M), 1 µl of dNTP (10 mM), 5 µl each of forward and reverse primers (10 µM), 0.5 µl of *Bst* DNA polymerase, along with plasmid template DNA/cDNA and nuclease-free water (NFW) to achieve the final volume.

Optimization of the PSR conditions for PEV-G was performed by varying parameters such as temperature, reaction time, forward primer concentration, and reverse primer concentration. The optimal reaction temperature was identified by testing temperatures ranging from 60°C to 68°C, in 1°C increments, for 2 hours, using a known concentration of plasmid DNA as the template. The optimal reaction time was evaluated by conducting the assay for 1, 1.5, 2, and 2.5 hours. Additionally, the optimal concentration ranges for both forward (1–4 µM) and reverse primers (1–4 µM) were determined using the checkerboard titration method, which involved testing a total of 16 combinations.

### Comparison of RPA and PSR assays with PCR

The RPA plasmid DNA was utilized in PCR reactions for comparison with the developed RPA assay. The RPA primers were used in a concentration of 0.48 µM forward and 0.48 µM reverse primer for performing conventional PCR. The cDNA prepared from field samples was used as a template in PCR reaction. The thermal conditions of PCR were finally optimized as initial denaturation at 95°C for 2 min followed by 40 cycles of denaturation at 95°C for 1 min, annealing at 50°C for 30 s and extension at 72°C for 45s and final elongation at 72°C for 10 min.

The PSR plasmid DNA was utilized in PCR reactions for comparison with the developed PSR assay. The PSR primers were also used in a concentration of 0.48 µM forward and 0.48 µM reverse primer for performing conventional PCR. The cDNA prepared from field samples was used as a template in PCR reaction. The thermal conditions of PCR were finally optimized as initial denaturation at 95°C for 2 min followed by 40 cycles of denaturation at 95°C for 1 min, annealing at 60°C for 30s and extension at 72°C for 45s and final elongation at 72°C for 10 min.

### Analytical sensitivity of assays

The analytical sensitivity of the assay is the limit of detection, i.e., up to what dilution the assay can detect the target sequence. The sensitivity of each assay was evaluated using ten-fold serial dilution up to 10^th^ dilution (10^−1^ to 10^−10^) of respective positive controls. The copy number calculation was done using the quantified DNA concentration according to the following formula:


$$Copynumber=[AxNo]/[length(plasmid+insert\;size)\times 1\times 10^{9}\times 660]\mathrm{\backslash ]}$$

In this formula, A represents the DNA concentration in ng/μl.

No. is Avogadro’s number (6.022X10^23^).

Assuming average weight of a nucleotide base pair (bp) is 660 Daltons and number of copies of template in the sample can be estimated by multiplying with 1x10^9^ (conversion factor- ng).

### Analytical sensitivity determination using DNA binding dye

DNA binding dye PicoGreen® (Invitrogen) was used for the colorimetric detection of amplicons in each assay. The RPA and PSR reactions were performed using the serially diluted plasmid DNA and incubated at optimum temperature and time conditions, and the sensitivity was then checked using both the dyes. A total of 1 μl of PicoGreen® dye (1:10 diluted) was added to 10 μl of the RPA and PSR reaction amplicons and fluorescence was observed on UV transilluminator.

#### Analytical specificity of assays.

The analytical specificity of any assay refers to the ability of the assay to accurately identify and measure a specific target in the presence of other similar substances or components. It assesses how well the assay distinguishes the target from non-target molecules, minimizing false positives and ensuring that the signals detected are truly associated with the target of interest. High analytical specificity implies that the assay will consistently produce reliable and relevant results even when other potentially interfering substances are present.

The analytical specificity assessment was conducted by testing the assay against related viral pathogens of pigs. This involved using positive controls for Porcine Posavirus, Porcine Sapelovirus, Porcine Parvovirus, Porcine Circovirus, and Classical Swine Fever Virus, all of which were available in our laboratory. The detection of these viruses was carried out using each of the developed assays, and the results were subsequently visualized on a 2.5% agarose gel.

### Screening of samples

The developed assay was evaluated using field samples including 100 faecal samples and nasal swabs collected from diseased as well as healthy pigs in organized and unorganized farms in Haryana (29.0588° N, 76.0856° E). These samples were screened using the developed RT-RPA and RT-PSR assay (as described in material and methods section) as well as with conventional PCR. It is important to mention here that one of the confirmed positive samples (IND2019/ABT114) was exhausted and was not available for testing in RT-PSR assay. Hence for evaluation of RT-PSR assay 99 samples were used instead of 100.

The RPA primer in a concentration of 0.48 µM forward and 0.48 µM reverse primer were used for performing conventional RT-PCR. The cDNA prepared from field samples (as described methods section) was used as a template in PCR reaction. The thermal conditions of PCR were finally optimized as initial denaturation at 95°C for 2 min followed by 40 cycles of denaturation at 95°C for 1 min, annealing at 50°C (RPA primers)/ 56°C (PSR primers) for 30s and extension at 72°C for 45s and final elongation at 72°C for 10 min.

## Results

### Optimization of reaction parameters for RPA assay

PEV-G specific recombinant plasmid was amplified with the help of RPA primer set. The reaction conditions for the RPA were optimized at 41ºC for 20 minutes. Although RPA functioned at various temperatures (35ºC, 37ºC, 39ºC, and 41ºC), the intensity of the bands from the amplified products indicated that 41ºC was the optimal temperature (S1 Fig in S1 File). To optimize the reaction time, incubations were carried out at different intervals: 5, 10, 15, 20, 25, and 30 minutes, all at 41ºC. Amplification was detectable starting at 5 minutes, but the most significant amplification (clearly observable on the gel) occurred at 20 and 25 minutes. Therefore, 20 minutes was determined to be the optimal incubation time for RPA (S2 Fig in S1 File). For primer concentration, a combination of 0.96 µM for the forward primer and 0.72 µM for the reverse primer yielded the maximum amplification (S3 Fig in S1 File), making this combination optimal. Magnesium acetate (MgOAc) concentrations were evaluated between 10 mM and 20 mM, with amplification observed at all concentrations but no significant differences in band intensities. The best amplification was noted at a concentration of 16 mM, which was therefore selected as the optimum concentration for the PEV-G RPA assay (S4 Fig in S1 File). Although a concentration of 16 mM appears to yield better RPA product, additional contaminating bands are present. In contrast, these additional bands are missing at 14mM concentration of MgOAc. Such additional bands will increase the intensity of colour in visual detection and will affect the limit of detection of assay. Hence, for visual detection approach, 14 mM MgOAc may be considered optimum.

### Optimization of reaction parameters for PSR assay

To optimize the temperature for PSR, the reaction mixture containing recombinant plasmid of PEV-G was incubated at temperatures ranging from 60ºC to 68ºC in 1ºC increments. Although PSR functioned at 64ºC, 65ºC, and 66ºC, the intensity of the bands from the PSR amplified products indicated that 65ºC was the optimal temperature for PEV-G (S5 Fig in S1 File). For time optimization, the PSR reaction mixture was incubated at the standardized temperature of 65ºC for various intervals ranging from 1.0 to 2.5 hours, with increments of 30 minutes. Amplification was initially detectable after 1.5 hours, but maximum amplification (producing a ladder-like pattern) was observed at 2.5 hours. Therefore, 2.5 hours was established as the optimal reaction time for PEV-G PSR (S6 Fig in S1 File).The optimal concentrations of both forward and reverse primers were determined using the checkerboard method, which involved creating 16 different combinations of primers. In this method, one variable (reverse primer concentration) was kept constant while gradually increasing the concentration of the other variable (forward primer concentration) in increments of 5. The combination of 1 µM forward primer and 3 µM reverse primer yielded the maximum amplification, making this combination optimal for PEV-G PSR (S7 Fig in S1 File).

### Analytical sensitivity of both assays

**Fig 1:**
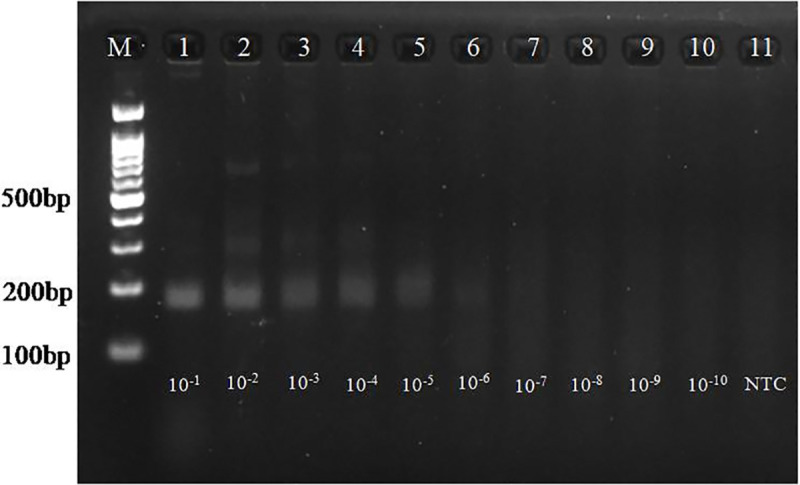
Analytical sensitivity of RPA reaction. Lane M: 100 bp ladder, L11: NTC, L1-L10: Serial 10-fold dilution of PEV-G plasmid DNA (10^−1^ to 10^−10^). The amplification was detected upto 6^th^ dilution which corresponded to 1.417 x 10^4^ copies.

The RPA assay was optimized at a constant temperature of 41°C for 20 minutes with forward and reverse primer concentration 0.96 μM and 0.72 μM respectively and with 16 mM magnesium acetate concentration. For PEV-G, the analytical sensitivity of both assays was evaluated using ten-fold serial dilutions of positive control plasmid DNA, ranging from 10^−1^ to 10^−10^. The concentration of the undiluted plasmid used for RPA assay was 490 ng/μl. In the RPA assay, amplicons were clearly detectable up to the 6^th^ dilution, corresponding to approximately 1.417 x 10^4^ copies (Fig 1). The non-template control exhibited no amplification. Conventional PCR detected plasmid DNA dilutions up to 1.417 x 10^7^ copies (upto 3^rd^ dilution), demonstrating lower sensitivity of PCR compared to the developed isothermal RPA assay, which could detect 1.417 × 10⁴ copies (up to the 6^th^ dilution) (S8 Fig in S1 File).

**Fig 2:**
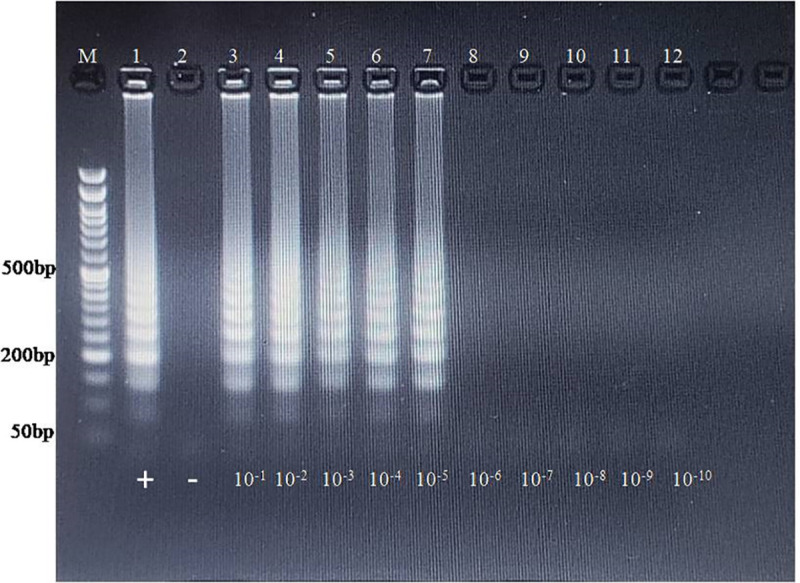
Analytical sensitivity of PSR reaction. Lane M: 100bp ladder, L11: NTC, L1-L10: Serial 10-fold dilution of PEV-G plasmid DNA (10^-1^ to 10^-10^). The amplification was detected upto 5^th^ dilution which corresponds to 2.3 x 10^5^copies.

The PSR assay was optimized at 65°C for 2.5 hr with forward and reverse primer concentration 1 μM and 3 μM respectively. For the PSR assay, the concentration of the undiluted plasmid for PSR assay was 81.5 ng/μl. The amplicons were clearly detectable on the gel up to the 5^th^ dilution, which corresponded to about 2.3 x 10^5^copies (Fig 2). As with the RPA assay, the non-template control showed no amplification. The same series of plasmid DNA dilutions was tested in conventional PCR for comparative analysis with the developed PSR assay. In this case, conventional PCR achieved amplification up to the 8^th^ dilution (230 copies), indicating that the conventional PCR assay is more sensitive than the newly developed isothermal PSR assay (S9 Fig in S1 File).

The analytical sensitivity was also determined using a DNA binding dye. Both assays were visually assessed using serial dilutions of plasmid DNA ranging from 10^-1^ to 10^-10^, with the aid of Picogreen dye. In these analysis, negative results appeared colourless, while positive results exhibited green fluorescence.

**Fig 3:**
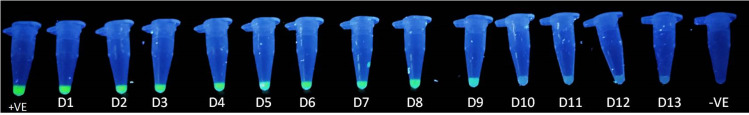
Visual detection of PEV-G RPA amplicons with different dilutions of plasmid DNA (10^-1^ to 10^-13^) using PicoGreen dye. Negative is colourless, positive is showing green fluorescence and with the increase in dilution green fluorescence is decreasing. The green fluorescence was detectable up to 9^th^ dilution and therefore the limit of visual detection was up to 14 copies.

For the RPA assay, visual detection revealed significant green fluorescence up to the 9^th^ dilution, establishing a limit of detection of approximately 14 copies (Fig 3). Although there was detection of some colour upto 13^th^ dilution compared to colourless NTC, which could be attributed to the presence of residual bacterial genomic DNA co-purified with the recombinant plasmids used as the synthetic positive control.

**Fig 4:**
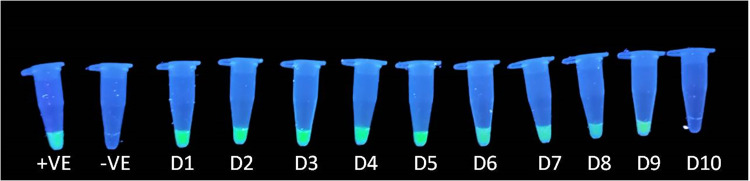
Visual detection of PEV-G PSR product with different dilutions of plasmid DNA (10^−1^ to 10^−10^) using PicoGreen dye. Negative is colorless, positive is green and with the increase in dilution green fluorescence is decreasing. The green fluorescence was detectable up to 9^th^ dilution and therefore the limit of detection was up to 2.3 copies.

In the case of the PSR assay, green fluorescence was detectable up to the 9^th^ dilution, corresponding to a limit of detection of around 2.3 copies (Fig.4).

### Analytical specificity of assays

**Fig 5:**
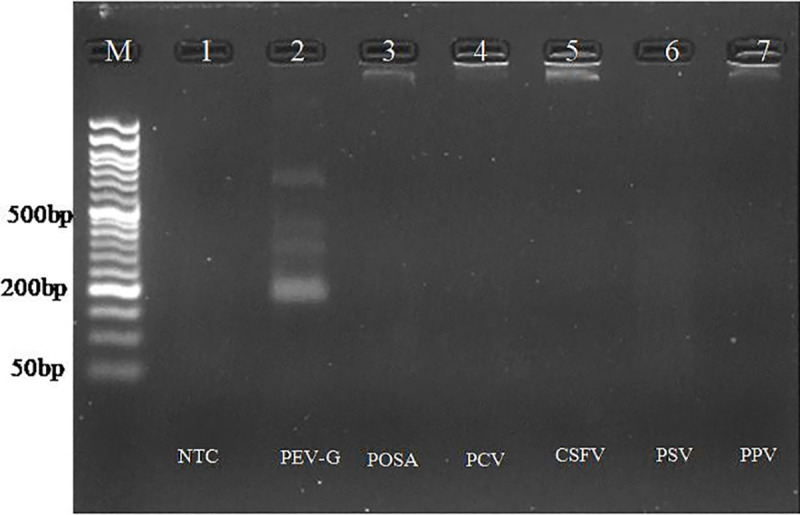
Analytical specificity of RPA assay. Lane M: 50 bp DNA ladder. L1: NTC, L2: RPA of PEV-G with optimized conditions. L3-L7: Heterologous reactions (POSA virus, PCV, CSFV, PSV, PPV). Amplification was only observed in lane 2 having nucleic acid from PEV-G.

**Fig 6:**
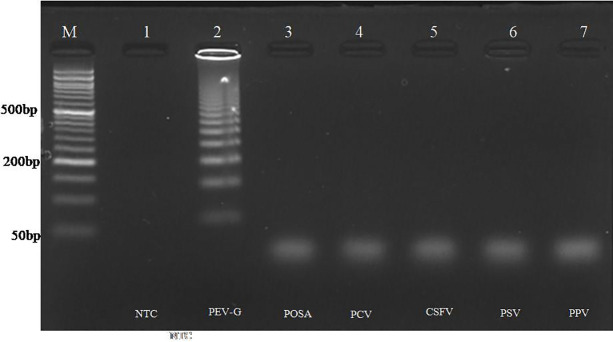
Analytical specificity of PSR assay. Lane M: 50 bp DNA ladder. L1: NTC, L2: PSR of PEV-G with optimized conditions. L3-L7: Heterologous reaction (POSA, PCV, CSFV, PSV, PPV). Amplification was only observed in lane 2 with nucleic acid from PEV-G.

To evaluate the specificity of both assays, testing was conducted with various related porcine viruses, including Classical Swine Fever Virus (CSFV), Porcine Posavirus (POSA), Porcine Sapelovirus (PSV), Porcine Parvovirus (PPV), and Porcine Circovirus (PCV). Amplification was detected solely with the PEV-G positive control, while no amplification was observed with the nucleic acids derived from the other viruses (Figs 5 and 6).

### Evaluation of developed assays

The developed assays were validated using 100 field samples collected from various pig farms. These samples were tested with the RT-RPA and RT-PSR assays, revealing that only three samples tested positive with the RT-RPA assay as confirmed by agarose gel electrophoresis (AGE) (S10 Fig in S1 File). The results from the RT-RPA assay were consistent with those obtained from RT-PCR testing and visual detection (S11 Fig in S1 File and S12 Fig in S1 File).

For RT-PSR assay only one sample was found positive by agarose gel electrophoresis (AGE) (S13 Fig in S1 File). While PCR results using primers developed for the RT-PSR assay indicated that two samples were positive (S14 Fig in S1 File). Additionally, dye analysis of the RT-PSR amplicon showed that two samples were positive (S15 Fig in S1 File). It is important to mention here that one of the confirmed positive samples (IND2019/ABT114) was exhausted and was not available for testing in RT-PSR assay. Hence for evaluation of RT-PSR assay 99 samples were used instead of 100. The data obtained showed that the results of both PCRs, RT-RPA and RT-PSR were consistent.

The developed one-step RT-RPA assay was also evaluated using PEV-G positive field samples, employing SuperScript™ IV and MMLV Reverse Transcriptase enzymes at 41°C for 20 minutes. While AGE did not show any visible RT-RPA products, green fluorescence was detected under UV light after the addition of Picogreen dye, indicating successful amplification of the target PEV-G RNA. No fluorescence was observed in the no-template control (NTC) (S16 Fig in S1 File) Validation through conventional RT-PCR demonstrated a distinct 179 bp band on a 2.5% agarose gel, confirming the presence of PEV-G RNA in both samples.

## Discussion

Isothermal nucleic acid amplification techniques have become increasingly valuable in the field of diagnostics, especially for viral infections such as Porcine Enterovirus-G (PEV-G). Traditional molecular diagnostic tools, such as RT-PCR and qPCR, offer high sensitivity and specificity but require expensive equipment and skilled personnel, making them unsuitable for resource-limited or field settings. In contrast, isothermal methods like Recombinase Polymerase Amplification (RPA) and Polymerase Spiral Reaction (PSR) operate under constant temperature conditions, providing simple, rapid, and cost-effective alternatives that are particularly beneficial in veterinary diagnostics and fieldwork. These methods not only offer a quicker turnaround time but also eliminate the need for thermal cyclers, making them ideal for point-of-care (POC) applications.

RPA primers, like PCR primers, are designed with specific considerations, including primer length. Earlier research suggested that RPA typically required longer primers, around 30–35 bp [19]. However, later studies have shown that shorter primers can also be effective. For example, in the development of an isothermal assay for detecting porcine epidemic diarrhea virus (PEDV), a primer pair targeting the PEDV N gene sequence was used, with both forward and reverse primers of 22 nucleotides [20]. In the present study, the focus was on developing and optimizing an RPA assay for Porcine Enterovirus-G (PEV-G), with primers designed to target the polyprotein region encoding non-structural proteins 2A and 3B. The forward primer was 22 bp, and the reverse primer was 20 bp, amplifying a 179 bp segment of the viral genome.

Reaction temperature is critical for RPA efficiency, with an optimal range of 37–42°C [21]. Previous studies identified optimal temperatures of 37°C for Coxsackievirus A6 [22], 39°C for African swine fever virus [23], and 42°C for Peste des petits ruminants virus (PPRV) detection [24]. In our study, 41°C was determined as the optimal incubation temperature for PEV-G detection. Compared to PCR and LAMP, RPA provides faster results, often achieving optimal amplification within 30 minutes [25] and sometimes as quickly as 3–4 minutes [26]. In this study, the reaction time was optimized for 20 minutes, achieving maximum amplification within this period for detection of PEV-G.

Primer concentration significantly influences RPA efficiency. Studies optimizing primer concentrations for RPA suggest effective ranges from 0.24 µM to 0.6 µM [27] and 0.24 µM to 0.96 µM [28]. For PEV-G, 0.96 µM of forward primer and 0.72 µM of reverse primer were identified as optimum for RPA assay. Unlike PCR, RPA is chemically initiated using magnesium acetate (MgOAc). Optimal MgOAc concentrations in previous studies ranged from 0.85 mM to 14 mM [29], and in this study, 16 mM concentration was optimized for PEV-G RPA assay.

For Polymerase spiral reaction (PSR), primers were designed to detect PEV-G by targeting the polyprotein region of the genome. Unlike LAMP, PSR uses only a single primer pair. An adapter sequence of exogenous origin was added at the 5′ end of both primers, ensuring the melting temperature (Tm) of the adapter sequence was 5°C lower than that of the primer [30]. For PEV-G PSR assay, primers of 39 bp (forward) and 38 bp (reverse) amplified a 195 bp polyprotein region of the PEV-G viral genome.

Optimization of reaction temperature for PEV-G PSR assay, 65°C was the optimal temperature which aligns with earlier studies conducted for detection of *Vibrio parahaemolyticus* [31] and Porcine Circovirus Type 3 [32]. The reaction time for PEV-G PSR assay was optimized to 2.5 hours, with amplification assessed by agarose gel electrophoresis (AGE). In previous study, for optimization of RT-PSR have been done for detection of New Castle disease virus at 65°C for 2hr [33].

Optimization of primer concentrations for RT-PSR assays has been explored for various viruses, including SARS-CoV-2 [34] and West Nile Virus [35], where optimal primer concentrations were respectively 1.6 µM and 4 µM. In the present study, we determined optimal primer concentrations of 1 µM (forward) and 3 µM (reverse).

In previous studies, RT-RPA assay and RT-RPA-LFS assay has been developed for Coxsackievirus A6 with limit of detection 101 copies/reaction and 102 copies/reaction respectively [22]. RT-RPA assay can detect TCSV RNA upto 10^−4^ dilution which was ten times more sensitive than RT-PCR (10^–3^) based on the results visualized on the AGE [36]. The findings of the present study are consistent with previous studies that have highlighted the superior sensitivity of RPA compared to PCR. The PEV-G RPA assay successfully detected up to 1.417 × 10^4^ copies (6^th^ dilution), surpassing conventional PCR’s detection limit of 1.417 × 10^7^copies (3^rd^ dilution).

Previous RT-PSR studies have reported detection limits for Astrovirus was 34.7 copies/µl [32] and for West Nile Virus was 1 RNA copy [35]. The PSR assay displayed comparable robustness, with sensitivity enabling the detection of PEV-G at a 10^5^ dilution (2.3 × 10^5^ copies), while conventional PCR reached a 10^−8^ dilution (2.3 × 10^2^ copies). Hence, the developed RPA and PSR assays demonstrated exceptional sensitivity and specificity for detecting porcine enterovirus-G (PEV-G), establishing them as reliable alternatives to conventional PCR.

The analytical specificity results have been observed in assays developed for other pathogens, such as Astrovirus in goslings [32], West Nile Virus [35], and SARS-CoV-2 [36]. Specificity analysis confirmed that RPA and PSR primers exclusively amplified the PEV-G genome without cross-reactivity with related viruses such as POSA virus, PSV, PCV, PPV, and CSFV, demonstrating its diagnostic precision.

Visual detection of both the RPA and PSR assays was achieved using the DNA intercalating dye Picogreen®. During amplification, the insoluble magnesium pyrophosphate products formed at the end of the isothermal process enable the direct visual detection of the reaction’s result due to the dye complex, providing an easy-to-read colored format for point-of-care (POC) testing [37,38]. Previous studies, where fluorescence of the amplicons decreased as the plasmid dilution increased [39]. Earlier studies have also utilized various dye-based detection methods for identifying recombinant plasmids of porcine sapelovirus [28] and *Salmonella* spp. [32].

RPA and PSR amplification for PEV-G was visible on an agarose gel up to the sixth (1.417 × 10⁴ copies) and 5^th^ dilution (2.3 x 10^5^ copies), respectively. While a fluorescence change in picogreen dye was observed up to the ninth dilution both in RPA (14 copies) and PSR (2.3 copies).

Isothermal assay-based screening of field samples has been reported earlier such as for PCV 3 virus [40] and bovine coronavirus [41]. Previously, porcine enterovirus-G was detected in porcine population in India [42]. Archived nasal and fecal swabs (n = 100) collected from pigs in Haryana were screened for PEV-G using the developed RT-RPA and RT-PSR assays alongside conventional RT-PCR. The RT-RPA assay identified three PEV-G-positive samples, confirmed via RT-PCR and visual detection using Picogreen dye.

In this study, the MMLV reverse transcriptase (RT) demonstrated better results compared to SuperScript™ IV (SSIV) showing higher amplification efficiency for PEV-G RNA. While both enzymes performed well, MMLV RT provided more consistent and robust amplification under the conditions used. In contrast, SSIV, although efficient at higher temperatures, did not achieve the same level of sensitivity/amplification in this specific assay. Therefore, MMLV RT was preferred for optimal performance in the RT-RPA assay. The RT-PSR assay detected two samples as positive although, one of the confirmed positive samples (IND2019/ABT114) was exhausted and could not be tested in RT-PSR assay. The data obtained showed that the results of both PCRs, RT-RPA and RT-PSR were consistent.

These results underscore the reliability of RPA and PSR as efficient alternatives to conventional PCR for PEV-G detection in field samples.

## Conclusions

This study underscores the successful development and optimization of RPA and PSR assays as effective diagnostic tools for detecting Porcine Enterovirus-G (PEV-G) in pigs. Both methods exhibited high sensitivity and specificity, with RPA delivering rapid results within 20 minutes and PSR providing reliable detection at 65°C in 2.5 hours. These assays have outperformed conventional PCR in terms of suitability for field applications, presenting significant benefits for resource-limited settings and point-of-care diagnostics. Validation with field samples further established their reliability and comparability to standard molecular techniques. The findings highlight the potential of RPA and PSR as invaluable tools for the timely and accurate management of PEV-G infections, thereby contributing to enhanced disease control in porcine populations. This advancement in diagnostic capability represents a vital step toward improving veterinary care and ensuring better livestock health.

## Supporting information

S1 File S1 to S16 Fig. Supplementary figures. (DOCX)

S2 File S1_raw_images. (PDF)
