# Supplementary material for: Development of isothermal nucleic acid amplification technologies for rapid detection of Porcine Enterovirus-G
Source: PLoS One. 2025 Jul 2;20(7):e0326700. doi: 10.1371/journal.pone.0326700 (PMC12221045; doi:10.1371/journal.pone.0326700)
Supplement: S1 File — (DOCX) [file pone.0326700.s001.docx]

# Supporting information:

**S1 to S16 Fig: Supplementary Figures**

# ‘Development of isothermal nucleic acid amplification technologies for rapid detection of Porcine Enterovirus-G’

**Sarishti Kaushik, Sushila Maan*, Kanisht Batra, Swati Sindhu, Vijay Kadian, and Aman Kumar**

**
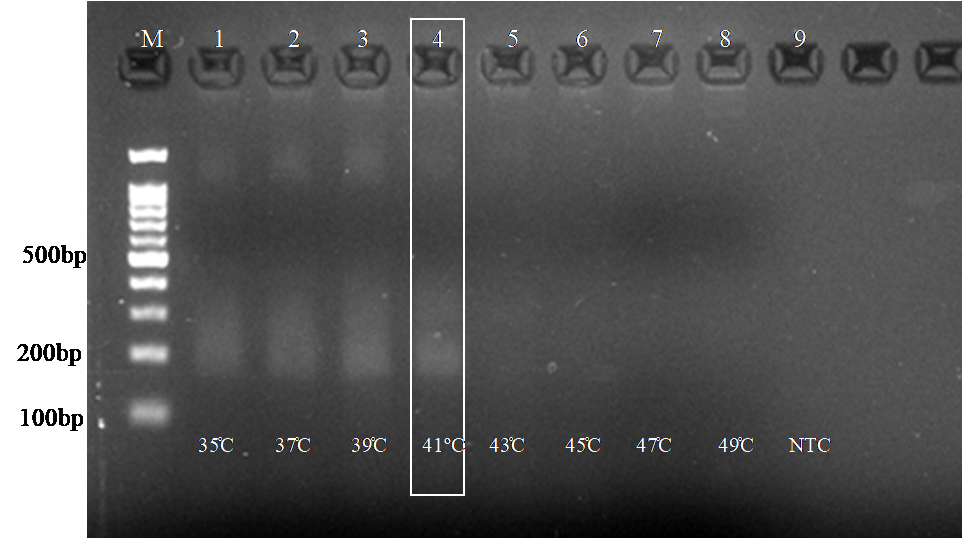
**

S1 Fig. Agarose gel electrophoresis of PEV-G RPA amplified product at variable temperatures on 2.5% gel. Lane M: 100bp DNA marker; Lane 9: NTC (non-template control); Lane 1-8: RPA amplified product at temperature 35ºC, 37ºC, 39ºC, 41ºC, 43ºC, 45ºC, 47ºC and 49ºC.The final optimized temperature for RPA was 41ºC.


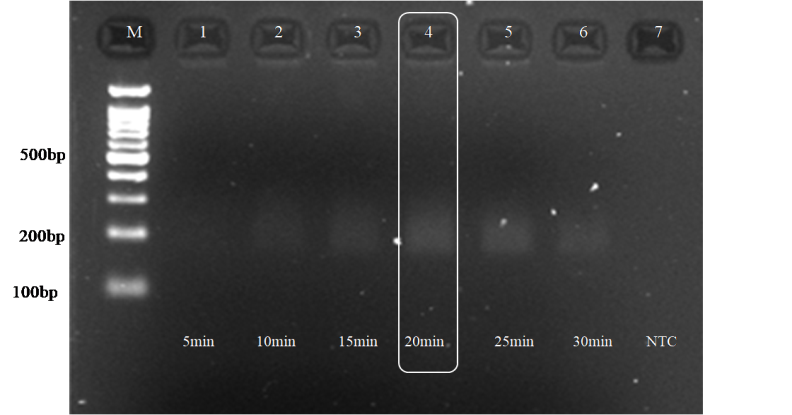


S2 Fig. Agarose gel electrophoresis of PEV-G RPA amplified product at variable time on 2.5% gel. Lane M: 100bp DNA marker; Lane 7: NTC (non-template control); Lane 1-6: RPA amplified product at time 5min, 10 min, 15 min, 20 min, 25 min and 30 min. The final optimized time for PEV-G RPA was 20 min.


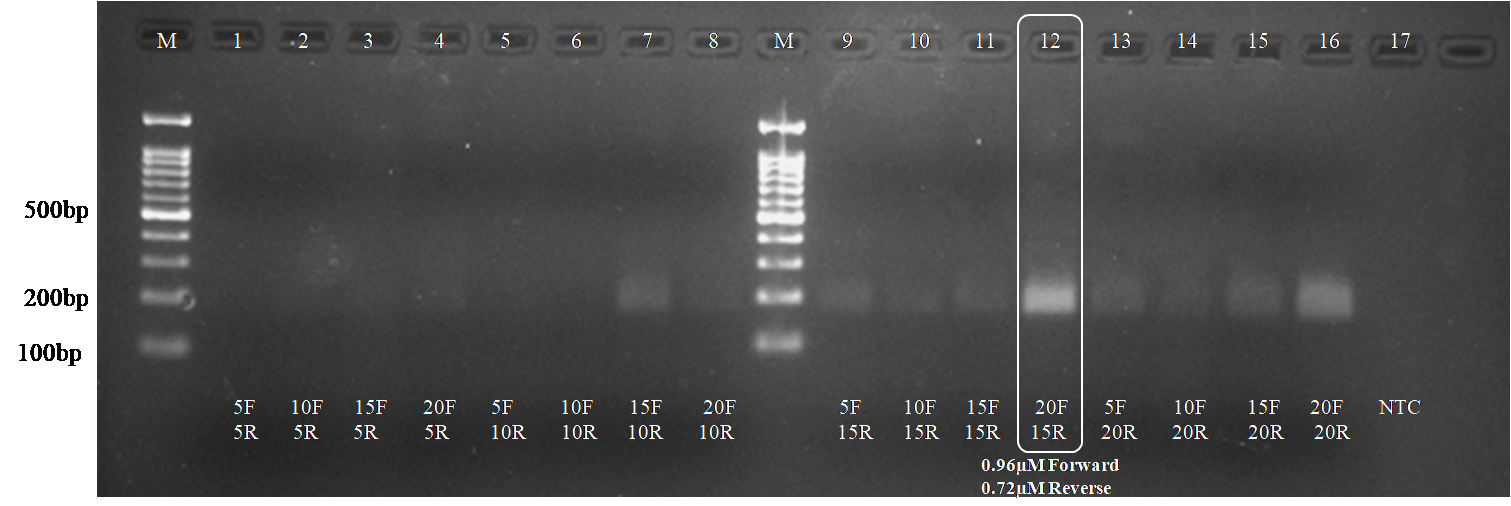


S3 Fig. Agarosegelelectrophoresis of PEV-G RPA reaction optimization for primer concentration using 2.5% gel. Lane M: 100bp ladder, L17: NTC, L1: 5F5R, L2: 10F5R, L3: 15F5R, L4: 20F5R, L5: 5F10R, L6: 10F10R, L7: 15F10R, L8: 20F10R, L9: 5F15R, L10: 10F15R, L11: 15F15R, L12: 20F15R, L13: 5F20R, L14: 10F20R, L15: 15F20R, L16: 20F20R.Final optimized primer concentration for PEV-G RPA reaction is 20F15Ri.e0.96μM of F primer and 0.72μM of R primer.

**
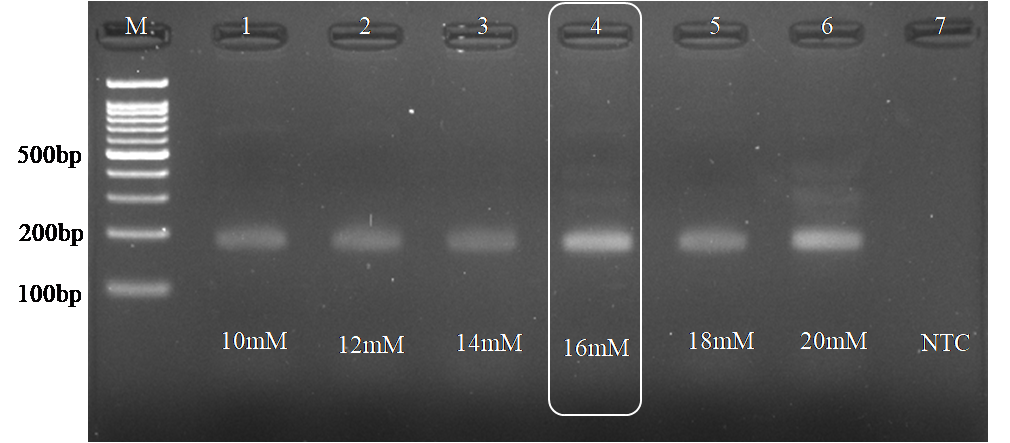
**

S4 Fig. Agarose gel electrophoresis of PEV-G RPA reaction for optimization of MgOAc concentration on 2.5% gel. Lane M: 100bp ladder, L7: NTC. L1-L6: Different MgOAc concentration of 10mM, 12mM, 14mM, 16mM, 18mM, 20mM.The final optimized MgOAc concentration was 16mM.


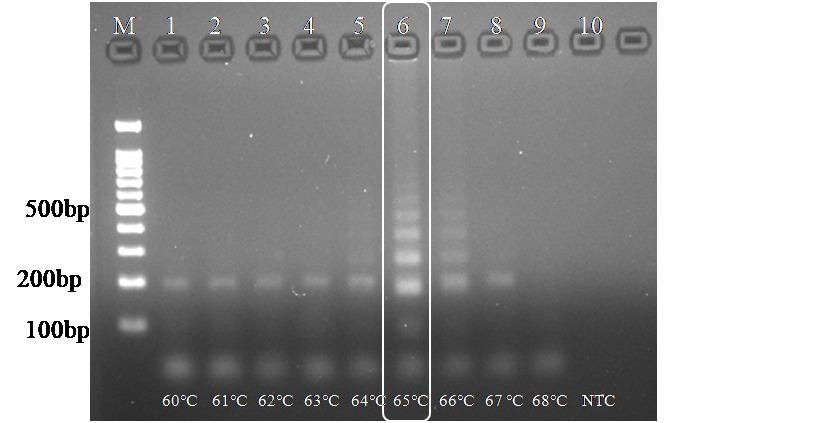


S5 Fig. Agarose gel electrophoresis of PEV-G PSR amplified product at variable temperatures. Lane M: 100 bp DNA marker; Lane 10: NTC; Lane 1-9: PSR amplified gene product at variable temperature (60℃, 61℃, 62℃, 63℃, 64℃, 65℃, 66℃ and 68℃).The final optimized temperature for PSR was 65℃.


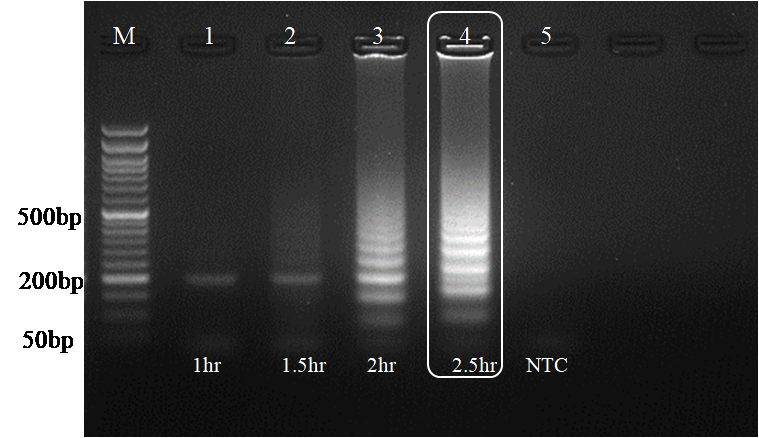


S6 Fig. Agarose gel electrophoresis of PEV-G PSR amplified product at variable time. Lane M: 50bp DNA marker; Lane 5: NTC; Lane 1-4: PSR amplified gene product at variable time 1hr, 1.5hr, 2hr, 2.5hr. The final optimized time for PEV-G PSR was 2.5hrs.


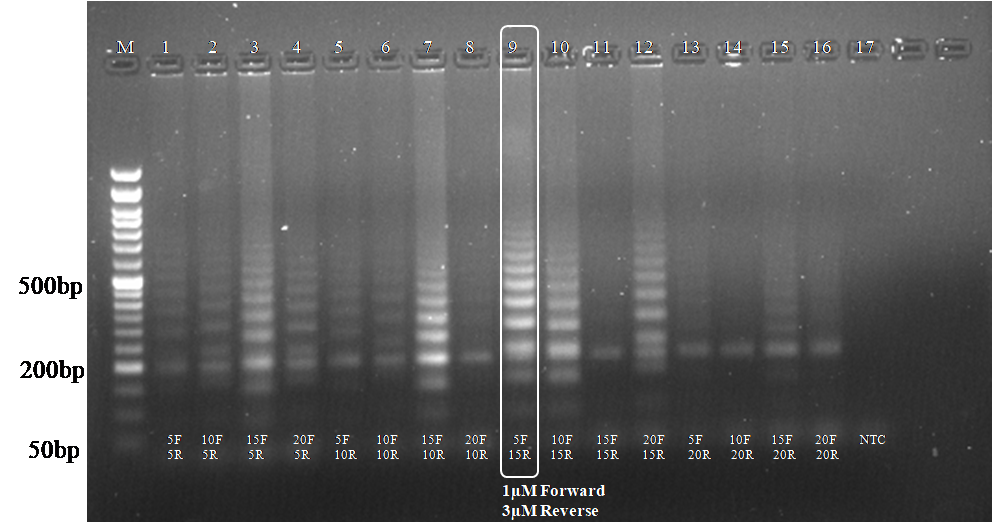


S7 Fig. Agarose gel electrophoresis of PEV-G PSR reaction optimization for primer concentration using 2.5% gel. Lane M: 50bp ladder, L17: NTC, L1: 5F5R, L2: 10F5R, L3: 15F5R, L4: 20F5R, L5: 5F10R, L6: 10F10R, L7: 15F10R, L8: 20F10R, L9: 5F15R, L10: 10F15R, L11: 15F15R, L12: 20F15R, L13: 5F20R, L14: 10F20R, L15: 15F20R, L16: 20F20R.Final optimized primer concentration for PEV-G PSR reaction is 5F15Ri.e.1μM of F primer and 3μM of R primer.

**
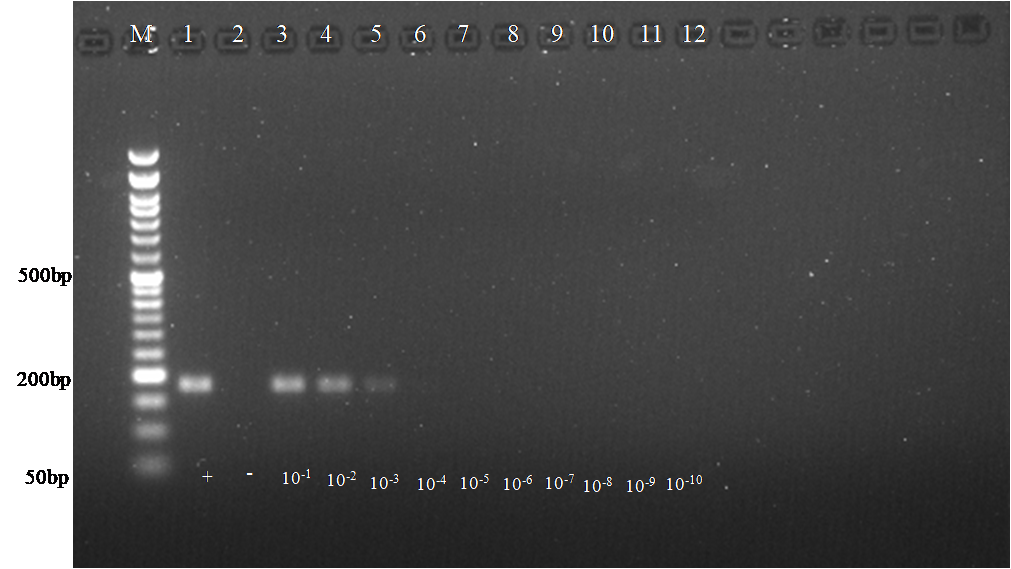
**

S8 Fig Agarose gel electrophoresis of PCR reaction with PEV-G RPA plasmid DNA dilutions on 2.5% gel. Lane M: 50bp ladder, L2: NTC, L1: Positive control. L3-L12: PEV-G Serial 10-fold dilution of plasmid DNA (10^-1^ to 10^-10^).

**
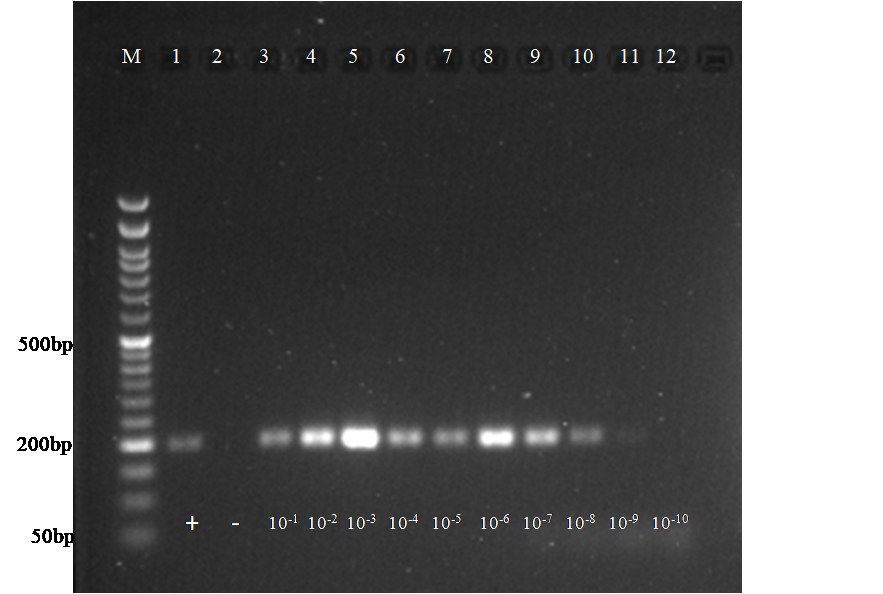
**

S9 Fig Agarose gel electrophoresis of PCR reaction with PSR PEV-G plasmid DNA dilutions on 2.5% gel. Lane M: 50bp ladder, L2: NTC. L1: Positive control. L3-L12: Serial 10-fold dilution of PEV-G plasmid DNA (10^-1^ to 10^-10^).


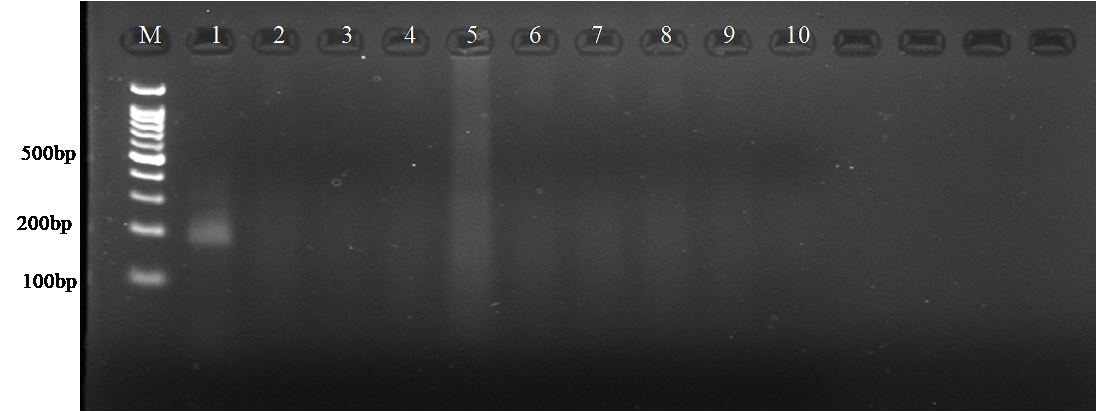


S10 Fig. Agarose gel electrophoresis (2.5%) showing results of screening of samples with developed RT-RPA for PEV-G. M: 100bp ladder, L1: PEV-G RPA positive control plasmid DNA, L2: NTC, L3: samples IND2021/ABT198, L4: samples IND2021/ABT278, L5: samples IND2019/ABT114, L6-10: random negative samples.


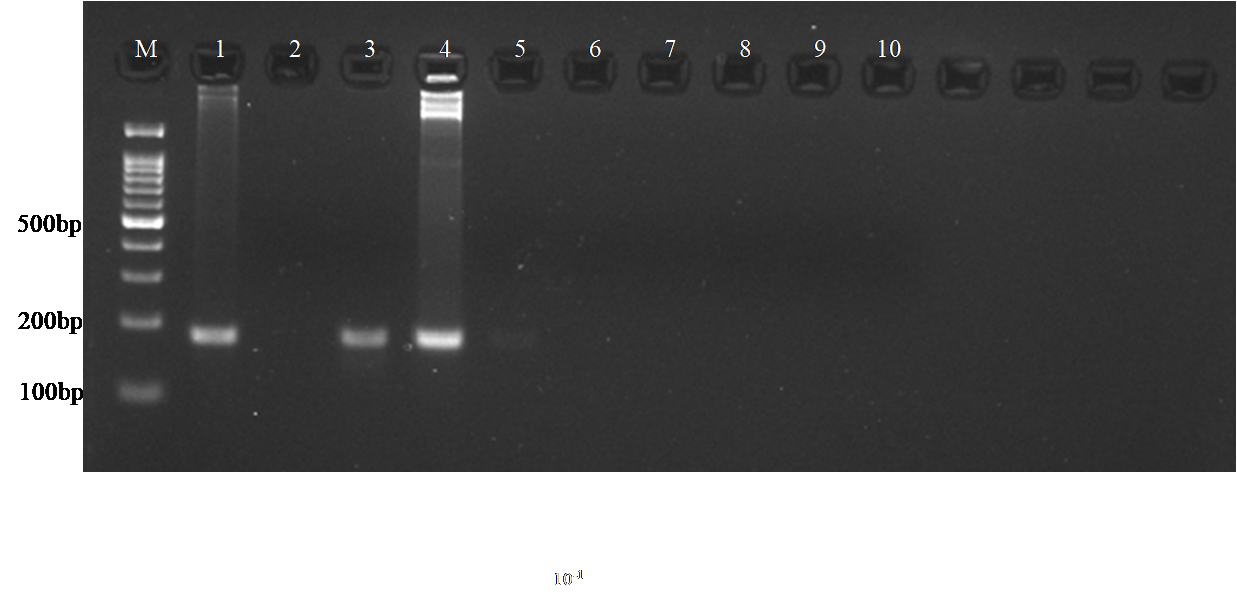


S11 Fig. Agarose gel electrophoresis (2.5%) showing results of screening of samples with conventional RT-PCR for PEV-G. M: 100bp ladder, L1: PEV-G RPA positive control plasmid DNA, L2: NTC, L3: sample IND2021/ABT198, L4: sample IND2021/ABT278, L5: sample IND2019/ABT114, L6-10: random negative samples.


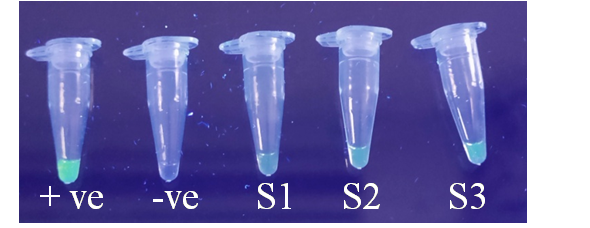


S12 Fig. Visual detection of PEV-G positive samples using PicoGreen dye. Negative are colourless, positive samples showing green fluorescence of variable intensity. Sample1: IND2021/ABT198, Sample2: IND2021/ABT278, Sample3: IND2019/ABT114.


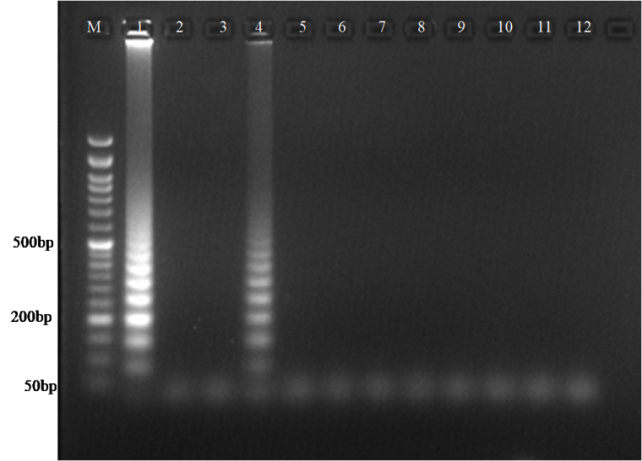


S13 Fig. Agarose gel electrophoresis (2.5%) showing results of screening of samples with developed RT-PSR for PEV-G. M: 50bp ladder, L1: PEV-G PSR positive control (with plasmid), L2: NTC, L3: samples IND2021/ABT198, L4: samples IND2021/ABT278, L5-10: random negative samples. Samples IND2021/ABT198 did not show ladder like pattern while sample IND2021/ABT278 showed typical ladder like pattern. Sample IND2019/ABT114 which was confirmed positive for PEV-G was not available for testing.


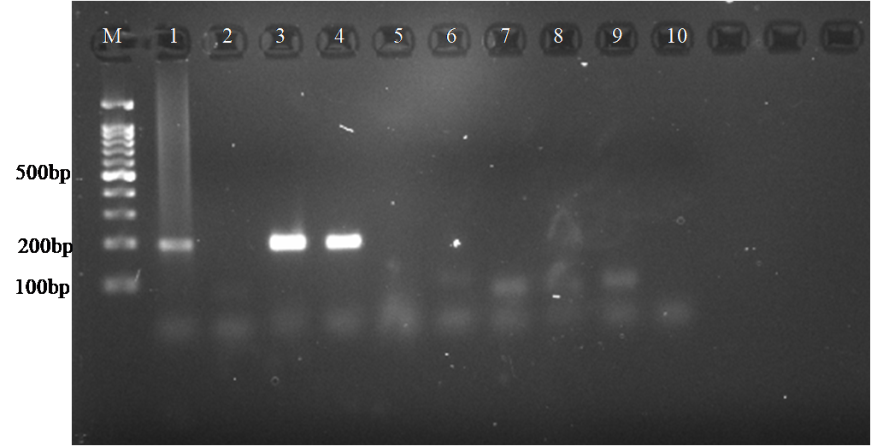


S14 Fig. Agarose gel electrophoresis (2.5%) showing results of screening of samples with conventional RT-PCR for PEV-G. M: 100bp ladder, L1: PEV-G PSR positive control (with plasmid), L2: NTC, L3: sample IND2021/ABT198, L4: sample IND2021/ABT278, L5-10: random negative samples. Both samples are positive in RT-PCR. Sample IND2019/ABT114 which was confirmed positive for PEV-G was not available for testing.


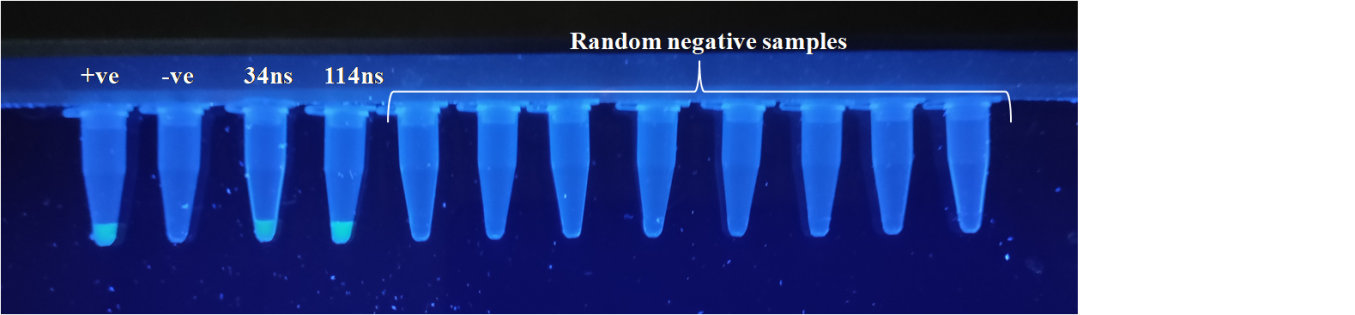


S15 Fig. Visual detection of PEV-G RT-PSR positive samples using PicoGreen dye. Negative are colorless, positive samples showing green fluorescence of variable intensity. Sample: IND2021/ABT198, Sample: IND2021/ABT278, Sample: IND2019/ABT114 which was confirmed positive for PEV-G was not available for testing.

**
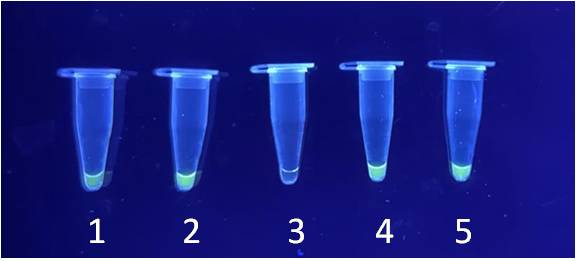
**

**S16 Fig. Visual detection of PEV-G samples with developed RT-RPA assay two different reverse transcriptase enzymes.**

Tube 1: PEV-G RPA with cDNA of sampleIND2021/ABT198using SuperScript™ IV Reverse Transcriptase,

Tube 2 : PEV-G RPA with cDNA of sampleIND2021/ABT278using SuperScript™ IV Reverse Transcriptase,

Tube 3 : NTC, Negative control did not any fluorescence under visual detection.

Tube 4 : PEV-G RPA with RNA of sampleIND2021/ABT198 amplified with MMLV RT.

Tube 5 : PEV-G RPA with RNA of sampleIND2021/ABT278 amplified with MMLV RT.
