# Supplementary material for: Development of isothermal nucleic acid amplification technologies for rapid detection of Porcine Enterovirus-G
Source: PLoS One. 2025 Jul 2;20(7):e0326700. doi: 10.1371/journal.pone.0326700 (PMC12221045; doi:10.1371/journal.pone.0326700)
Supplement: S2 File — (PDF) [file pone.0326700.s002.pdf]

Supporting information:

S1_raw_images

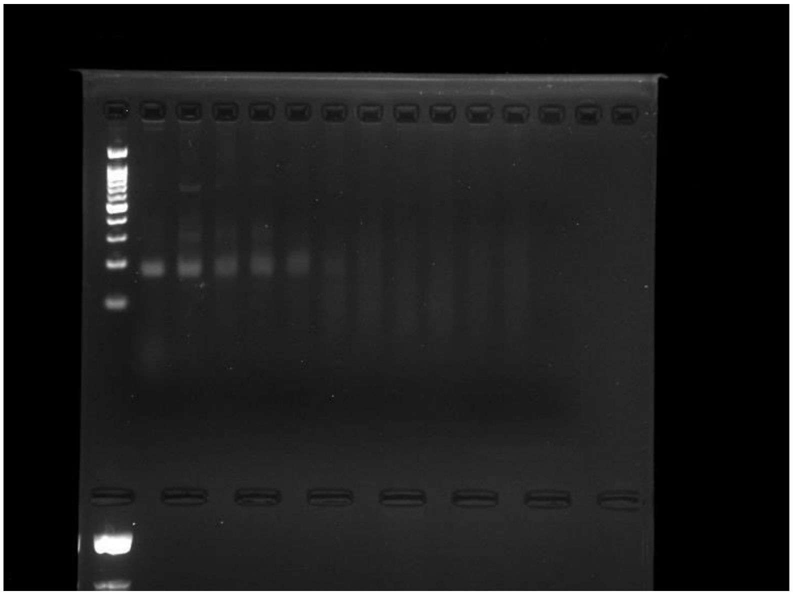

Fig 1: Analytical sensitivity of RPA reaction. Lane M: 100bp ladder, L11: NTC, L1-L10: Serial 10-fold dilution of PEV-G plasmid DNA (10^{-1} to 10^{-10}). The amplification was detected upto 6th dilution which corresponded to 1.417 x 10^4 copies.

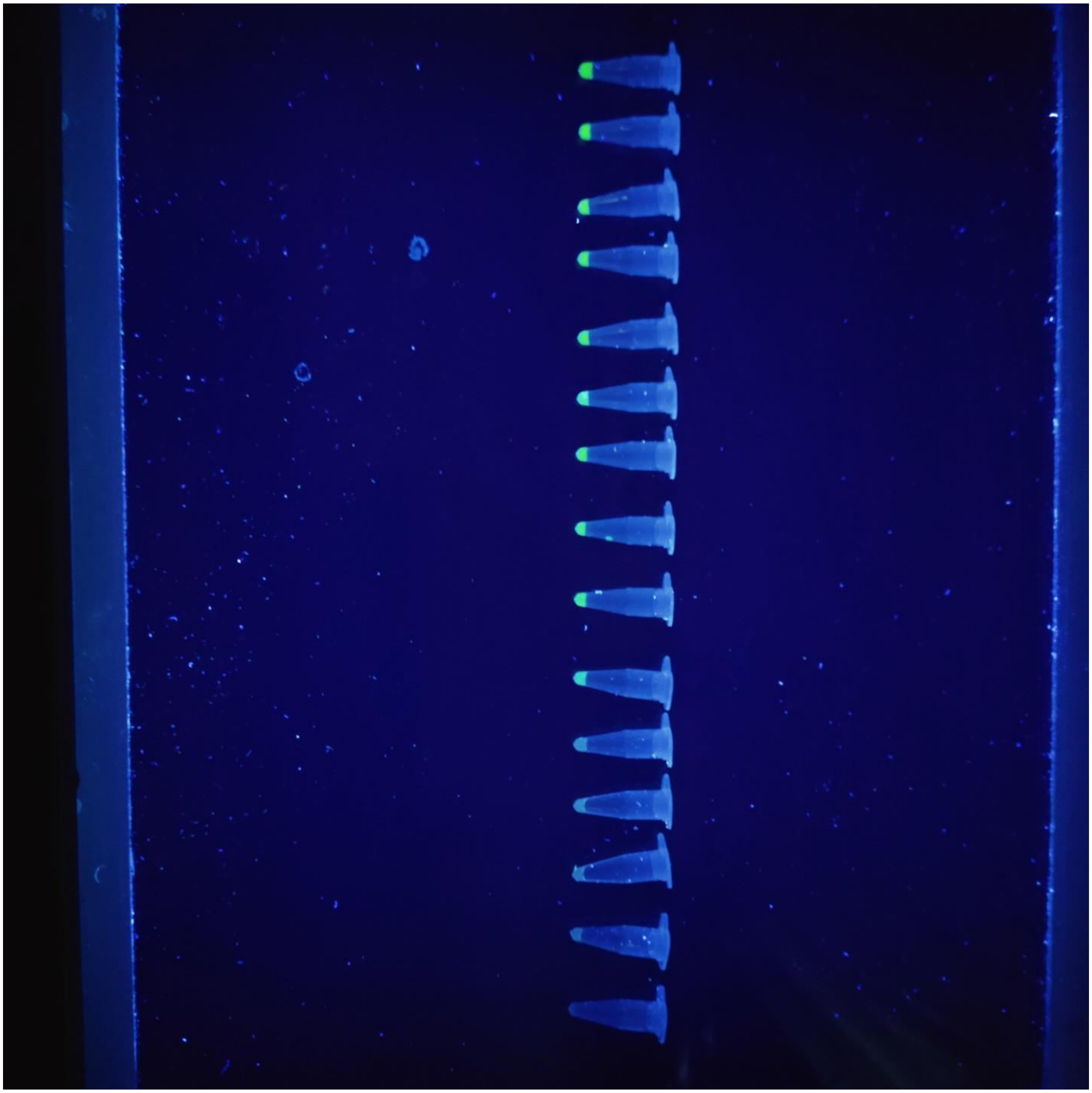

Fig 2: Visual detection of PEV-G RPA amplicons with different dilutions of plasmid DNA (10^{-1} to 10^{-13}) usingPicoGreen dye. Negative is colorless, positive is showing green fluorescence and with the increase in dilution green fluorescence is decreasing. The green fluorescence was detectable up to 11th dilution and therefore the limit of visual detection was up to 1.417 copies.

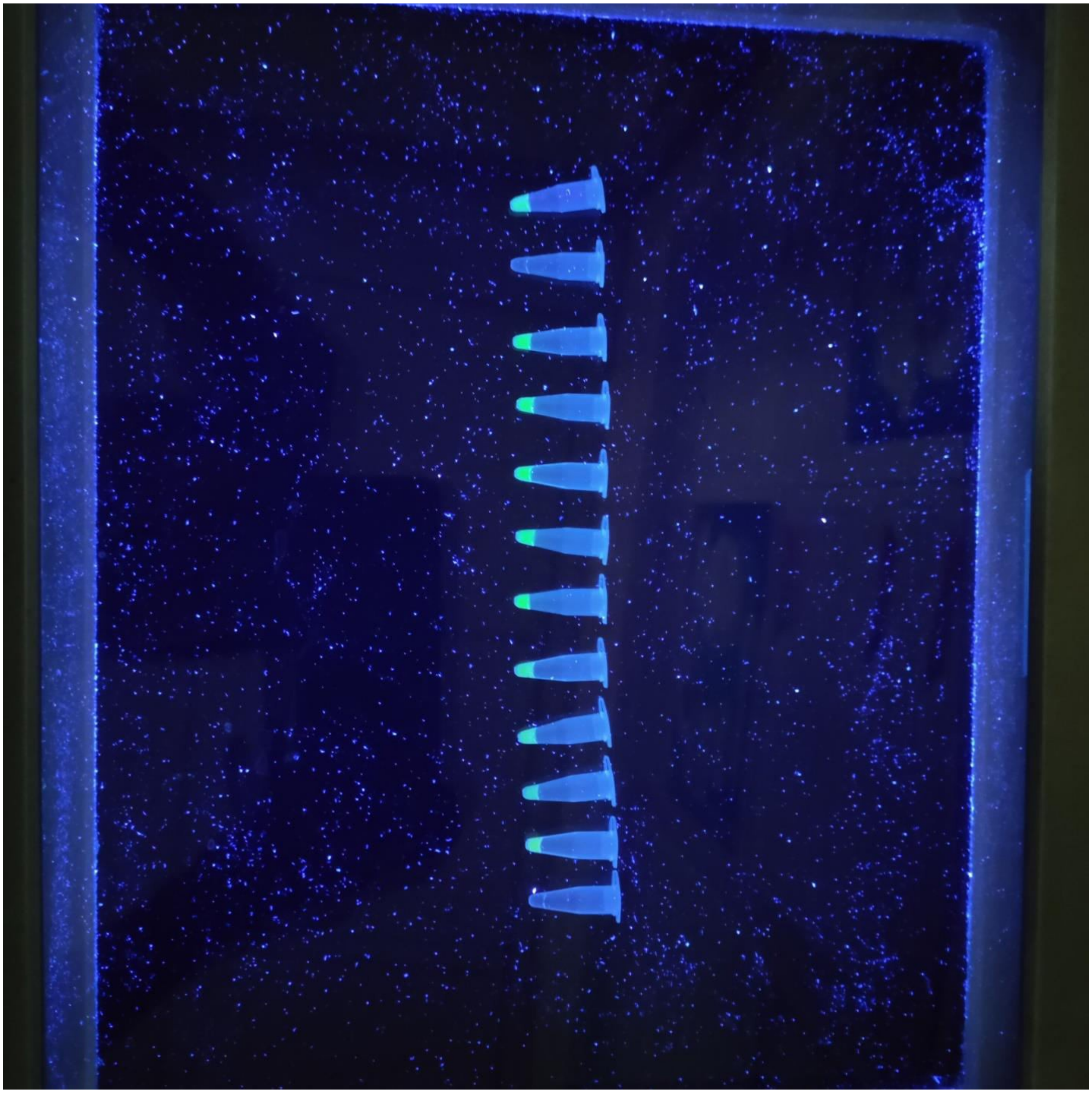

Fig 3: Visual detection of PEV-G PSR product with different dilutions of plasmid DNA (10^{-1} to 10^{-10}) usingPicoGreen dye. Negative is colorless, positive is green and with the increase in dilution green fluorescence is decreasing. The green fluorescence was detectable up to 9th dilution and therefore the limit of detection was up to 2.3 copies.

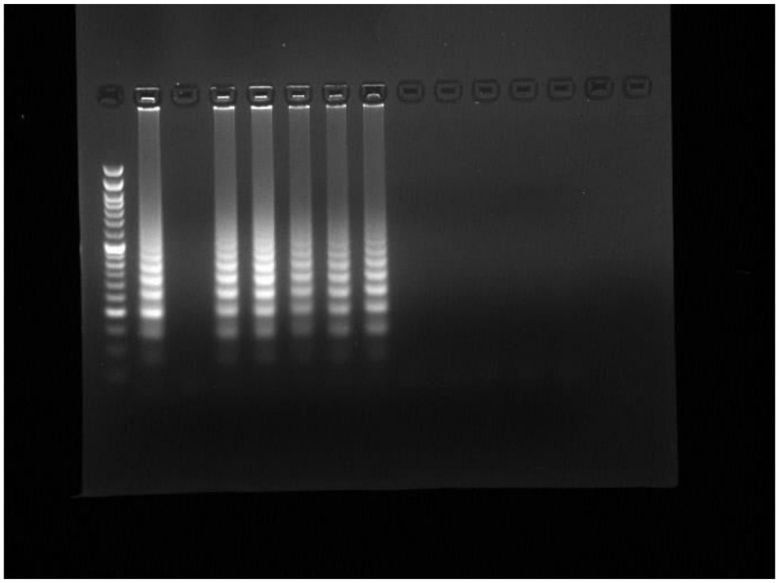

Fig 4: Analytical sensitivity of PSR reaction. Lane M: 100bp ladder, L11: NTC, L1-L10: Serial 10-fold dilution of PEV-G plasmid DNA (10^{-1} to 10^{-10}). The amplification was detected upto 5th dilution which corresponds to2.3 x 10^{5}copies.

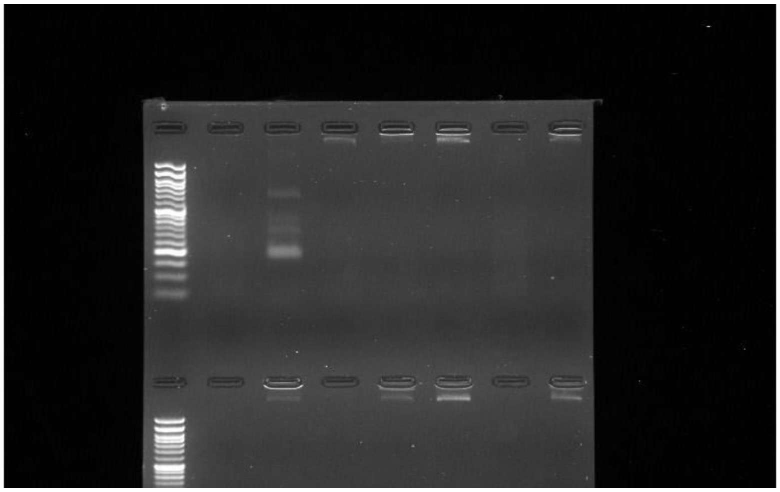

Fig 5: Analytical specificity of RPA assay. Lane M: 50 bp DNA ladder. L1: NTC, L2: RPA of PEV-G with optimized conditions. L3-L7: Heterologous reactions (POSA virus, PCV, CSFV, PSV, PPV). Amplification was only observed in lane 2 having nucleic acid from PEV-G.

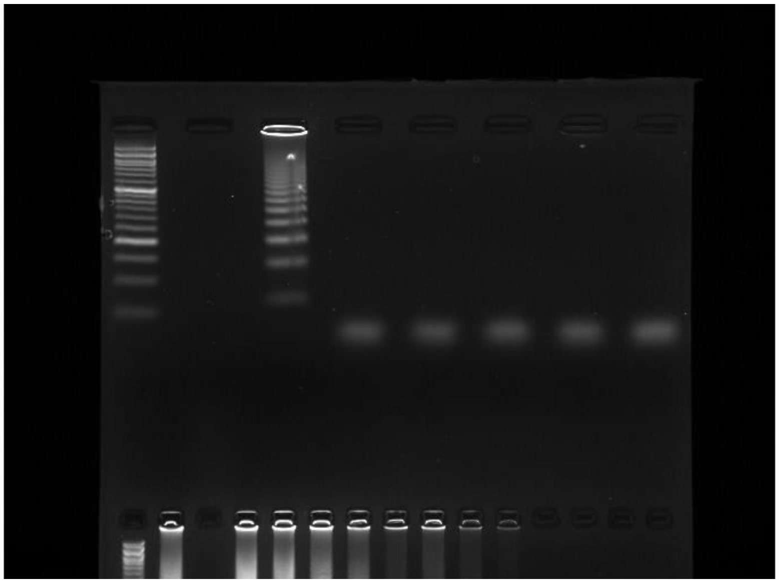

Fig 6: Analytical specificity of PSR assay. Lane M: 50 bp DNA ladder. L1: NTC, L2: PSR of PEV-G with optimized conditions. L3-L7: Heterologous reaction (POSA, PCV, CSFV, PSV, PPV). Amplification was only observed in lane 2 with nucleic acid from PEV-G

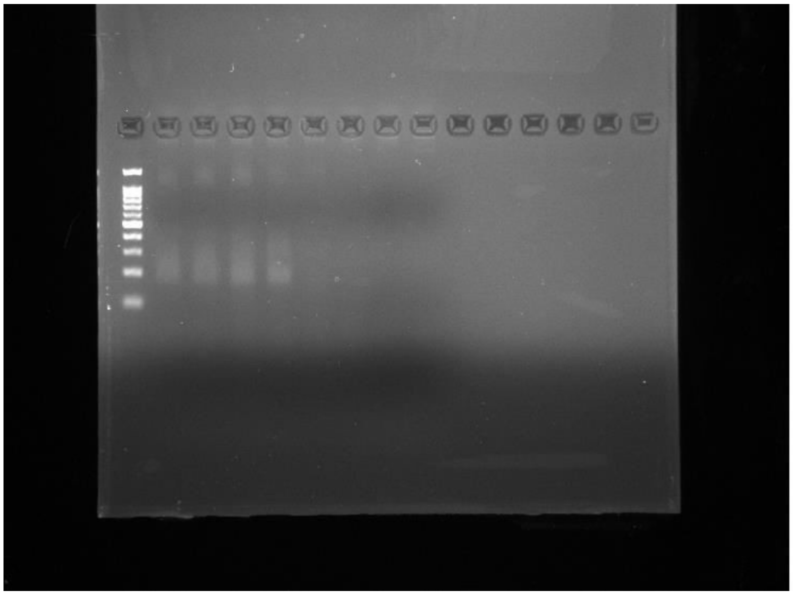

S1 Fig. Agarose gel electrophoresis of PEV-G RPA amplified product at variable temperatures on 2.5% gel. Lane M: 100bp DNA marker; Lane 9: NTC (non-template control); Lane 1-8: RPA

amplified product at temperature 35ºC, 37ºC, 39ºC, 41ºC, 43ºC, 45ºC, 47ºC and 49ºC.The final optimized temperature for RPA was 41ºC.

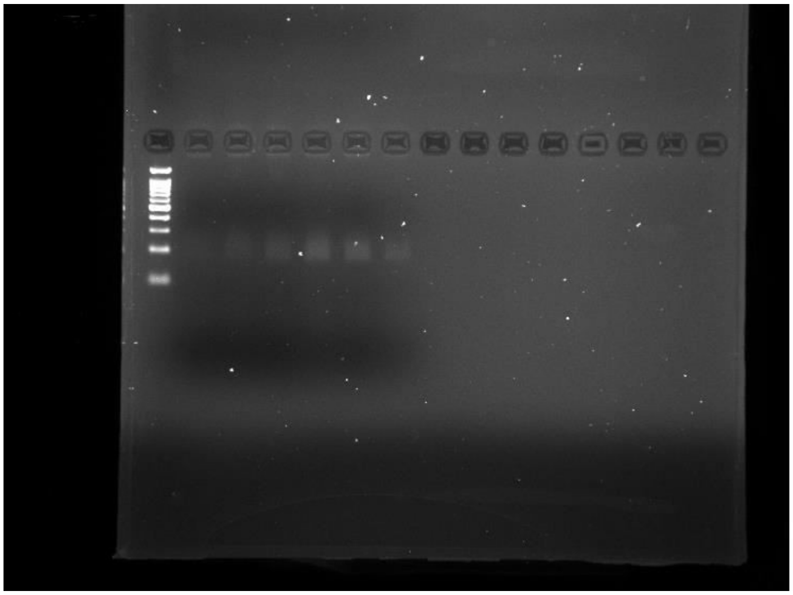

S2 Fig. Agarose gel electrophoresis of PEV-G RPA amplified product at variable time on 2.5% gel. Lane M: 100bp DNA marker; Lane 7: NTC (non-template control); Lane 1-6: RPA amplified product at time 5min, 10 min, 15 min, 20 min, 25 min and 30 min. The final optimized time for PEV-G RPA was 20 min.

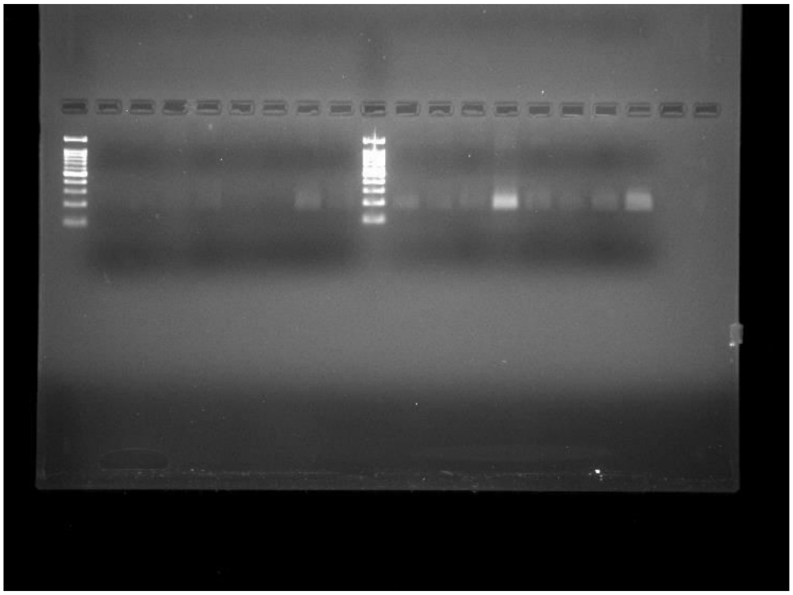

S3 Fig Agarose gel electrophoresis of PEV-G RPA reaction optimization for primer concentration using 2.5% gel. Lane M: 100bp ladder, L17: NTC, L1: 5F5R, L2: 10F5R, L3: 15F5R, L4: 20F5R, L5: 5F10R, L6: 10F10R, L7: 15F10R, L8: 20F10R, L9: 5F15R, L10: 10F15R, L11: 15F15R, L12: 20F15R, L13: 5F20R, L14: 10F20R, L15: 15F20R, L16: 20F20R.Final optimized primer concentration for PEV-G RPA reaction is 20F15Ri.e0.96µM of F primer and 0.72µM of R primer.

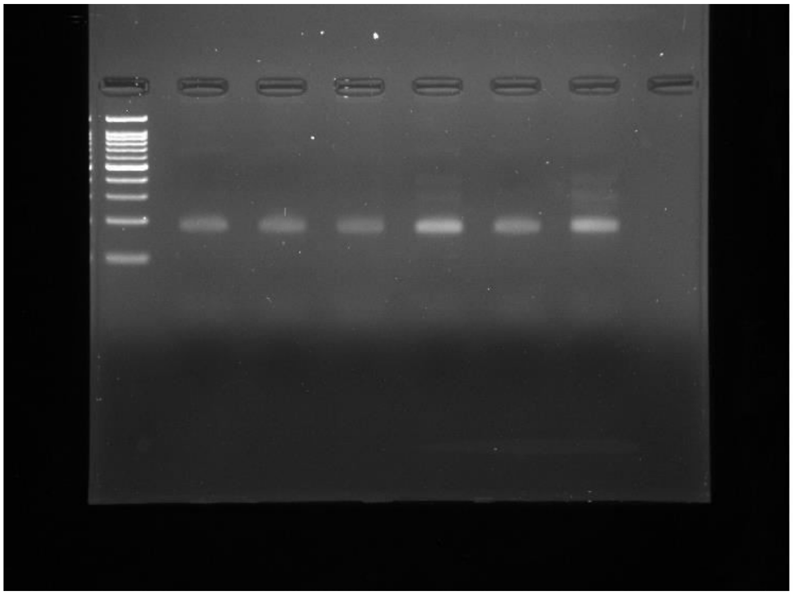

S4 Fig Agarose gel electrophoresis of PEV-G RPA reaction for optimization of MgOAc concentration on 2.5% gel. Lane M: 100bp ladder, L7: NTC. L1-L6: Different MgOAc concentration of 10mM, 12mM, 14mM, 16mM, 18mM, 20mM.The final optimized MgOAc concentration was 16mM.

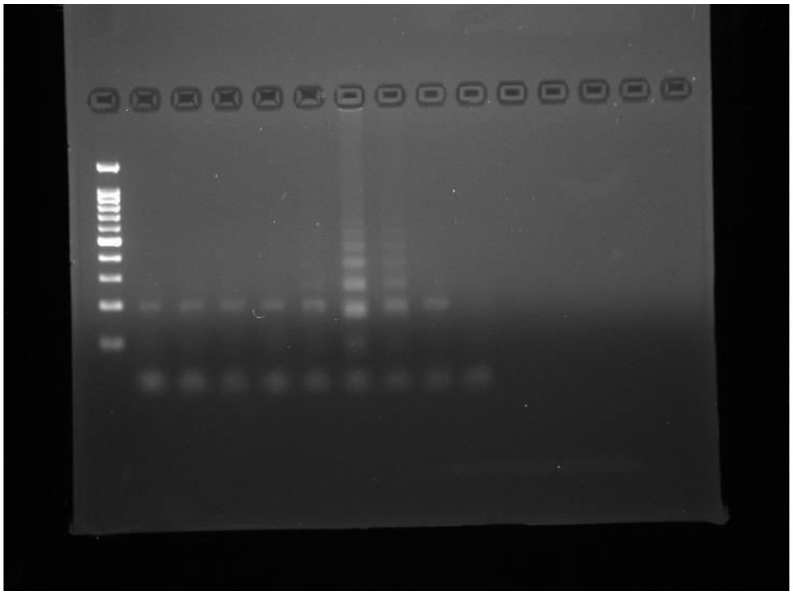

S5 Fig Agarose gel electrophoresis of PEV-G PSR amplified product at variable temperatures. Lane M: 100 bp DNA marker; Lane 10: NTC; Lane 1-9: PSR amplified gene product at variable temperature (60℃, 61℃, 62℃, 63℃, 64℃, 65℃, 66℃ and 68℃).The final optimized temperature for PSR was 65℃.

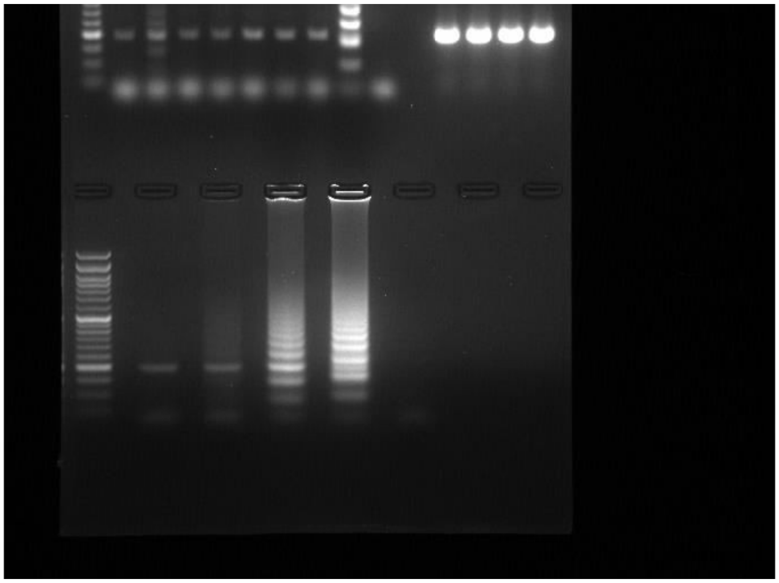

S6 Fig Agarose gel electrophoresis of PEV-G PSR amplified product at variable time. Lane M: 50bp DNA marker; Lane 5: NTC; Lane 1-4: PSR amplified gene product at variable time 1hr, 1.5hr, 2hr, 2.5hr. The final optimized time for PEV-G PSR was 2.5hrs.

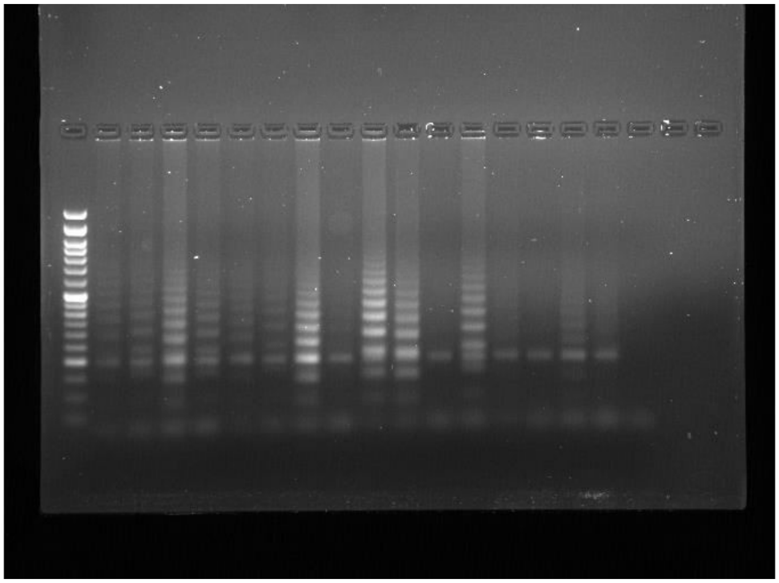

S7 Fig Agarose gel electrophoresis of PEV-G PSR reaction optimization for primer concentration using 2.5% gel. Lane M: 50bp ladder, L17: NTC, L1: 5F5R, L2: 10F5R, L3: 15F5R, L4: 20F5R, L5: 5F10R, L6: 10F10R, L7: 15F10R, L8: 20F10R, L9: 5F15R, L10: 10F15R, L11: 15F15R, L12: 20F15R, L13: 5F20R, L14: 10F20R, L15: 15F20R, L16: 20F20R.Final optimized primer concentration for PEV-G PSR reaction is 5F15Ri.e.1μM of F primer and 3μM of R primer.

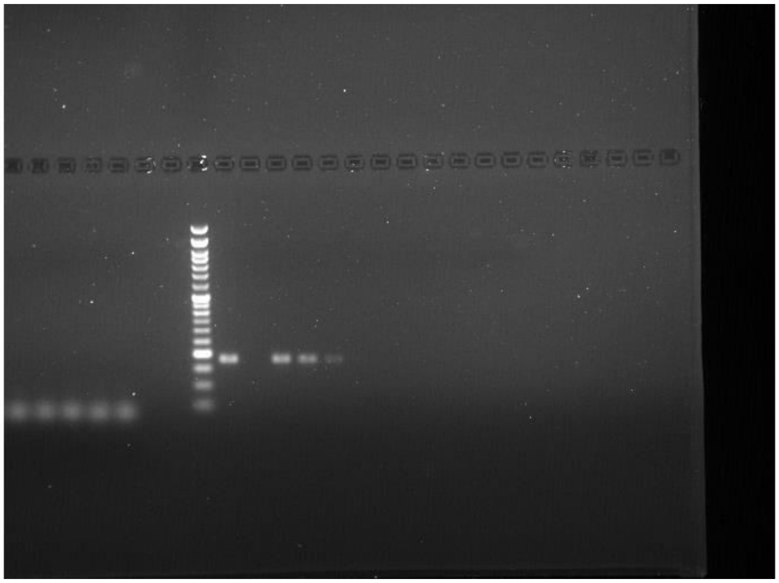

S8 Fig Agarose gel electrophoresis of PCR reaction with PEV-G RPA plasmid DNA dilutions on 2.5% gel. Lane M: 50bp ladder, L2: NTC, L1: Positive control. L3-L12: PEV-G Serial 10-fold dilution of plasmid DNA (10-1 to 10-10).

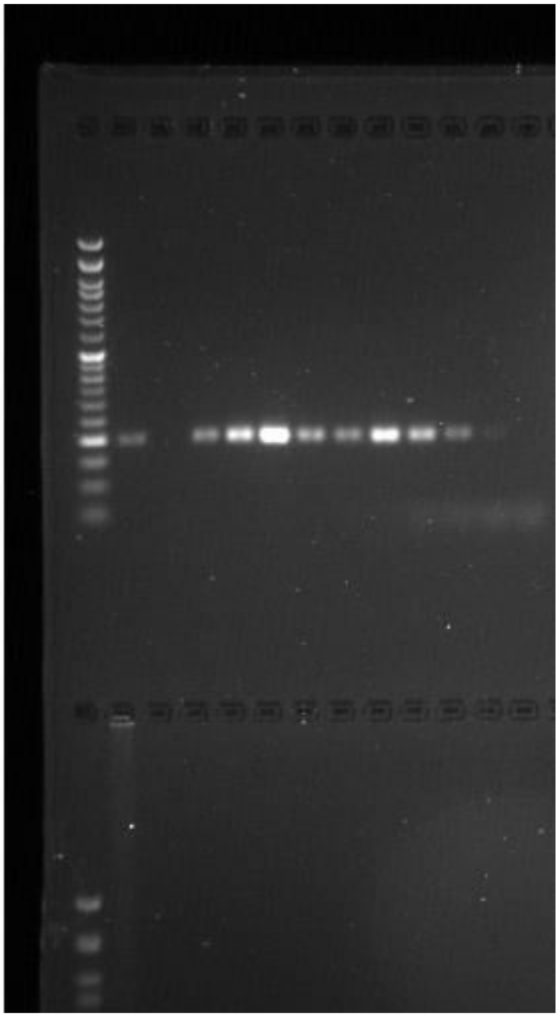

S9 Fig. Agarose gel electrophoresis of PCR reaction with PSR PEV-G plasmid DNA dilutions on 2.5% gel. Lane M: 50bp ladder, L2: NTC. L1: Positive control. L3-L12: Serial 10-fold dilution of PEV-G plasmid DNA (10^{-1} to 10^{-10}).

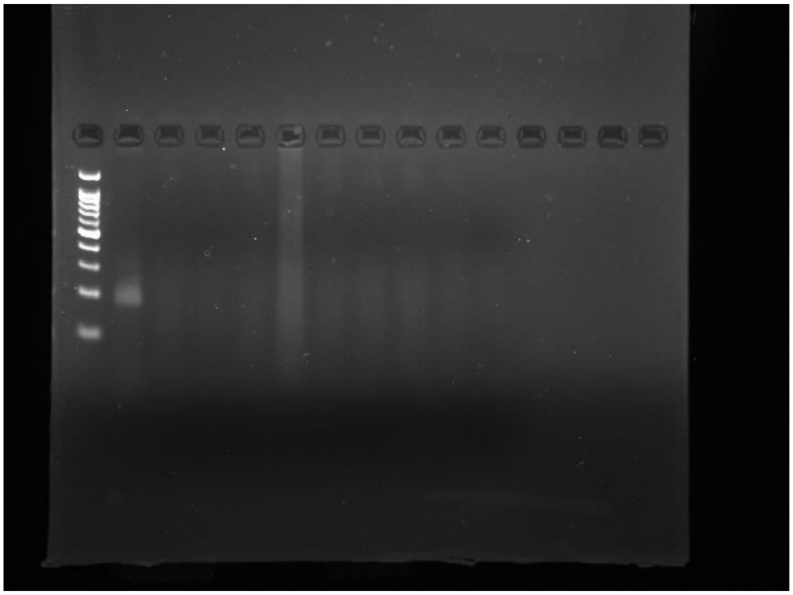

S10 Fig. Agarose gel electrophoresis (2.5%) showing results of screening of samples with developed RT-RPA for PEV-G. M: 100bp ladder, L1: PEV-G RPA positive control plasmid DNA, L2: NTC, L3: samples IND2021/ABT198, L4: samples IND2021/ABT278, L5: samples IND2019/ABT114, L6-10: random negative samples.

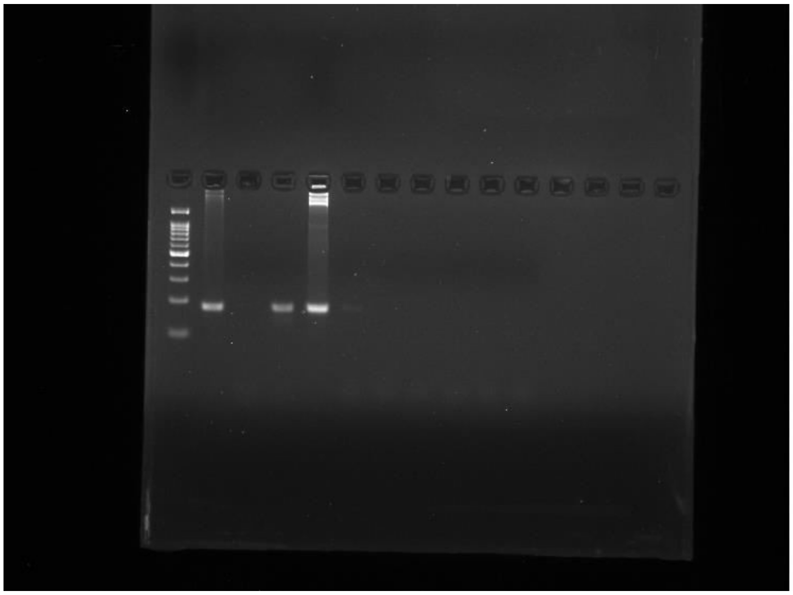

S11 Fig. Agarose gel electrophoresis (2.5%) showing results of screening of samples with conventional RT-PCR for PEV-G. M: 100bp ladder, L1: PEV-G RPA positive control plasmid

DNA, L2: NTC, L3: sample IND2021/ABT198, L4: sample IND2021/ABT278, L5: sample IND2019/ABT114, L6-10: random negative samples.

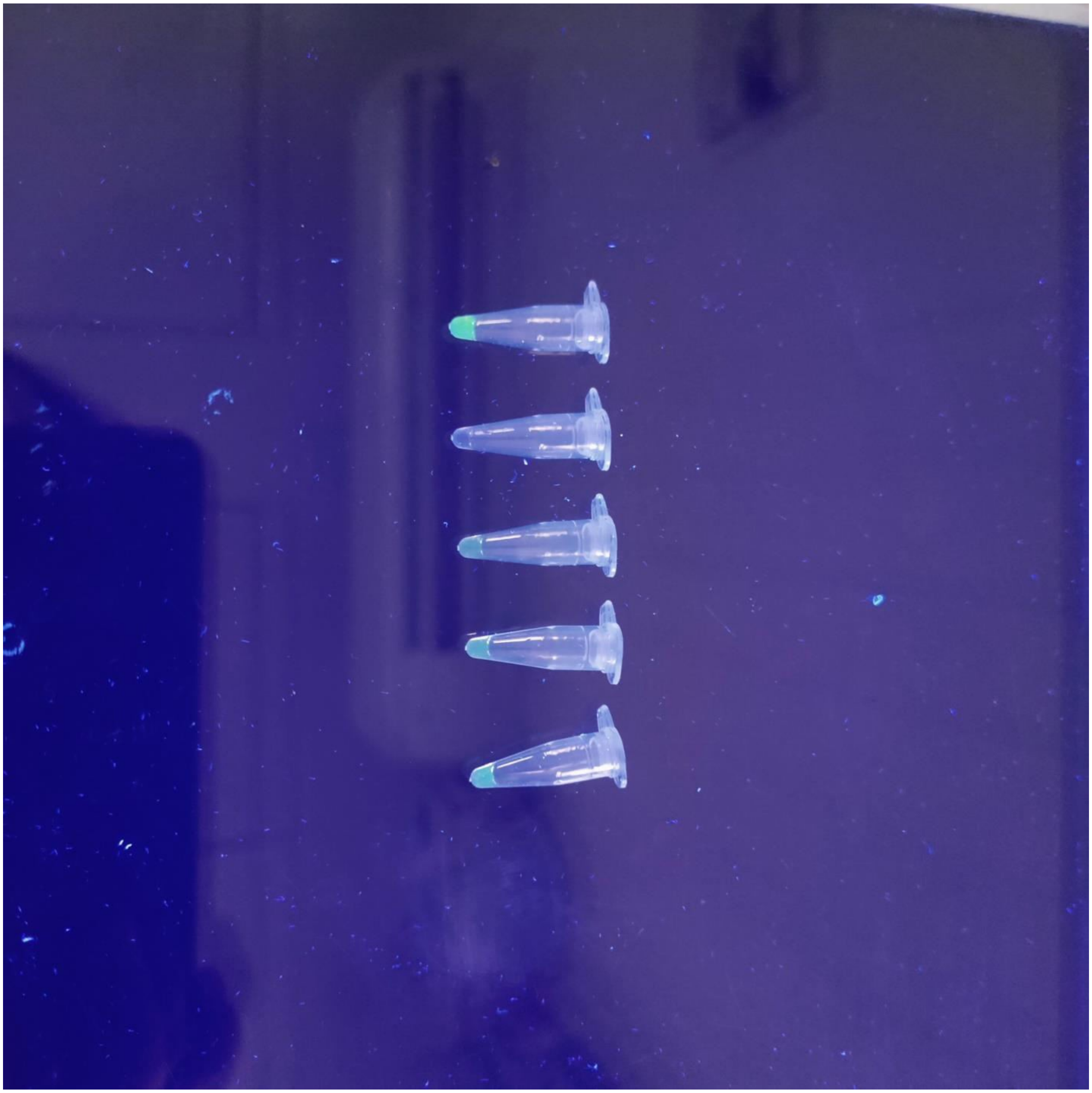

S12 Fig. Visual detection of PEV-G positive samples using PicoGreen dye. Negative are colourless, positive samples showing green fluorescence of variable intensity. Sample1: IND2021/ABT198, Sample2: IND2021/ABT278, Sample3: IND2019/ABT114.

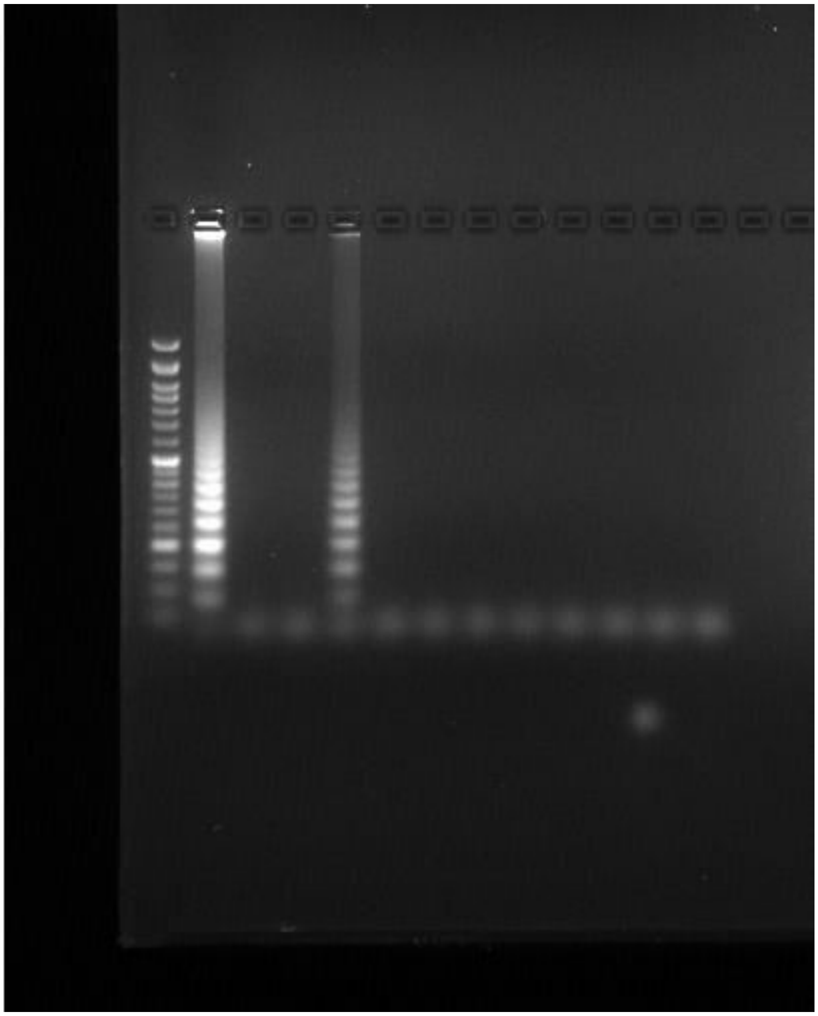

S13 Fig. Agarose gel electrophoresis (2.5%) showing results of screening of samples with developed RT-PSR for PEV-G. M: 50bp ladder, L1: PEV-G PSR positive control (with plasmid), L2: NTC, L3: samples IND2021/ABT198, L4: samples IND2021/ABT278, L5-10: random negative samples. Samples IND2021/ABT198 did not show ladder like pattern while sample IND2021/ABT278 showed typical ladder like pattern. Sample IND2019/ABT114 which was confirmed positive for PEV-G was not available for testing.

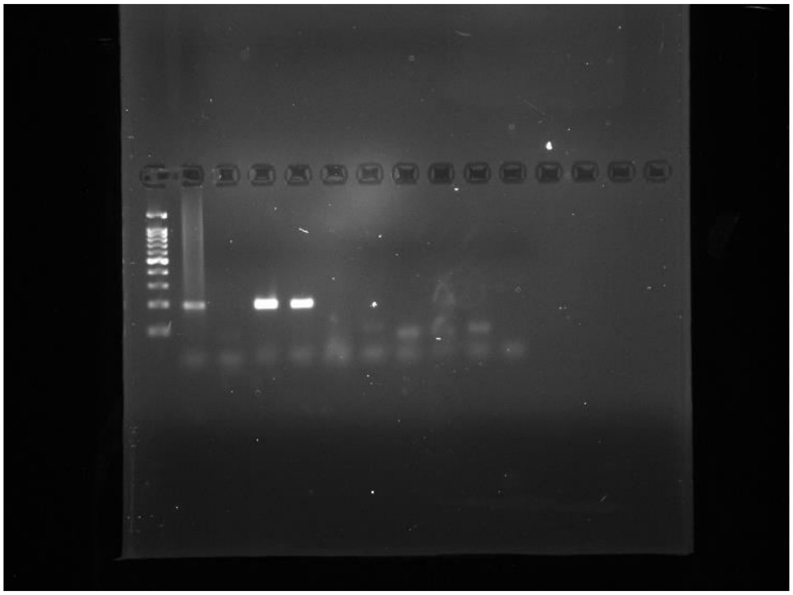

S14 Fig. Agarose gel electrophoresis (2.5%) showing results of screening of samples with conventional RT-PCR for PEV-G. M: 100bp ladder, L1: PEV-G PSR positive control (with plasmid), L2: NTC, L3: sample IND2021/ABT198, L4: sample IND2021/ABT278, L5-10: random negative samples. Both samples are positive in RT-PCR. Sample IND2019/ABT114 which was confirmed positive for PEV-G was not available for testing.

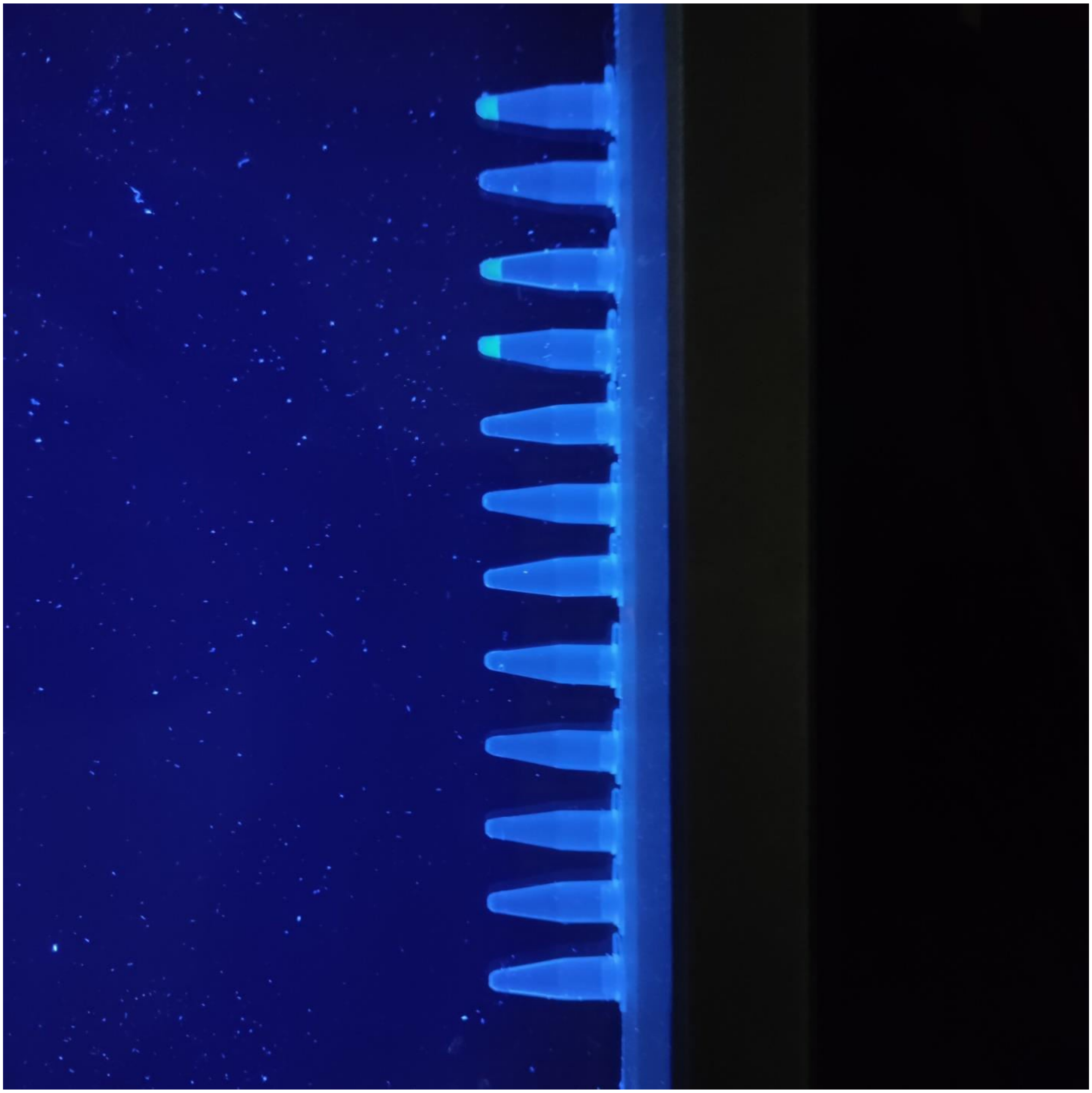

S15 Fig. Visual detection of PEV-G RT-PSR positive samples using PicoGreen dye. Negative are colorless, positive samples showing green fluorescence of variable intensity. Sample: IND2021/ABT198, Sample: IND2021/ABT278, Sample: IND2019/ABT114 which was confirmed positive for PEV-G was not available for testing.

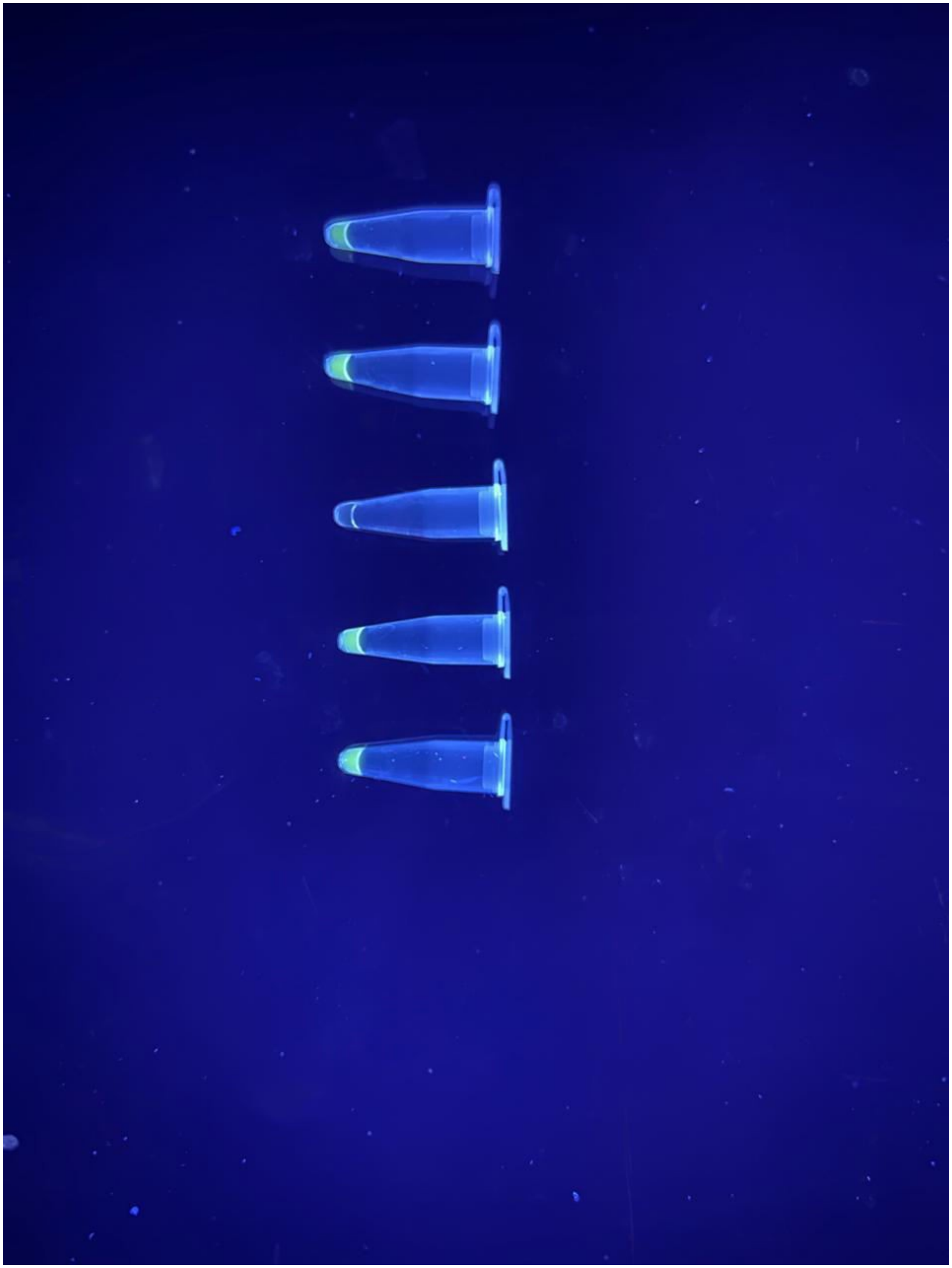

S16 Fig. Visual detection of PEV-G samples with developed RT-RPA assay two different reverse transcriptase enzymes.

Tube 1: PEV-G RPA with cDNA of sampleIND2021/ABT198using SuperScript™ IV Reverse Transcriptase,

Tube 2 : PEV-G RPA with cDNA of sampleIND2021/ABT278using SuperScript™ IV Reverse Transcriptase,

Tube 3 : NTC, Negative control did not any fluorescence under visual detection.

Tube 4 : PEV-G RPA with RNA of sampleIND2021/ABT198 amplified with MMLV RT.

Tube 5 : PEV-G RPA with RNA of sampleIND2021/ABT278 amplified with MMLV RT.
